# Immunogenicity of alternative ten-valent pneumococcal conjugate vaccine schedules in infants in Ho Chi Minh City, Vietnam: results from a single-blind, parallel-group, open-label, randomised, controlled trial

**DOI:** 10.1016/S1473-3099(20)30775-1

**Published:** 2021-10

**Authors:** Paul Vincent Licciardi, Beth Temple, Vo Thi Trang Dai, Nguyen Trong Toan, Doan Uyen, Cattram Duong Nguyen, Thanh V Phan, Kathryn Bright, Rachel Ann Marimla, Anne Balloch, Tran Ngoc Huu, Kim Mulholland

**Affiliations:** a New Vaccines Group, Murdoch Children's Research Institute, Melbourne, VIC, Australia; b Department of Paediatrics, University of Melbourne, Melbourne, VIC, Australia; c Global Health, Menzies School of Health Research, Charles Darwin University, Darwin, NT, Australia; d Epidemiology and Population Health, London School of Hygiene & Tropical Medicine, London, UK; e Microbiology and Immunology, Pasteur Institute of Ho Chi Minh City, Ho Chi Minh City, Vietnam; f Department of Disease Control and Prevention, Pasteur Institute of Ho Chi Minh City, Ho Chi Minh City, Vietnam

## Abstract

**Background:**

Data are scarce from low-income and middle-income countries (LMICs) to support the choice of vaccination schedule for the introduction of pneumococcal conjugate vaccines (PCV). We aimed to compare the immunogenicity of four different infant PCV10 schedules in infants in Vietnam.

**Methods:**

In this single-blind, parallel-group, open-label, randomised controlled trial, infants aged 2 months were recruited by community health staff in districts 4 and 7 of Ho Chi Minh City, Vietnam. Eligible infants had no clinically significant maternal or prenatal history and were born at or after 36 weeks' gestation. Participants were randomly assigned (3:3:5:4:5:4) using block randomisation, stratified by district, to one of six PCV10 or PCV13 vaccination schedules. Here we report results for four groups: group A, who were given PCV10 at ages 2, 3, 4, and 9 months (a 3 + 1 schedule); group B, who were vaccinated at ages 2, 3, and 4 months (3 + 0 schedule); group C, who were vaccinated at ages 2, 4, and 9·5 months (2 + 1 schedule); and group D, who were vaccinated at ages 2 and 6 months (two-dose schedule). Laboratory-based assessors were masked to group allocation. Blood samples were collected at different prespecified timepoints between ages 3–18 months depending on group allocation, within 27–43 days after vaccination, and these were analysed for serotype-specific IgG and opsonophagocytic responses. Participants were followed-up until age 24 months. The primary outcome was the proportion of infants with serotype-specific IgG levels of 0·35 μg/mL or higher at age 5 months, analysed as a non-inferiority comparison (10% margin) of the two-dose and three-dose primary series (group C *vs* groups A and B combined). We also compared responses 4 weeks after two doses administered at either ages 2 and 4 months (group C) or at ages 2 and 6 months (group D). The primary endpoint was analysed in the per-protocol population. Reactogenicity has been reported previously. This study is registered with ClinicalTrials.gov, NCT01953510, and is now closed to accrual.

**Findings:**

Between Sept 30, 2013, and Jan 9, 2015, 1201 infants were enrolled and randomly assigned to group A (n=152), group B (n=149), group C (n=250), group D (n=202), or groups E (n=251) and F (n=197). In groups A–D, 388 (52%) of 753 participants were female and 365 (48%) were male. 286 (95%) participants in groups A and B combined (three-dose primary series) and 237 (95%) in group C (two-dose primary series) completed the primary vaccination series and had blood samples taken within the specified time window at age 5 months (per-protocol population). At this timepoint, a two-dose primary series was non-inferior to a three-dose primary series for eight of ten vaccine serotypes; exceptions were 6B (84·6% [95% CI 79·9–88·6] of infants had protective IgG concentrations after three doses [groups A and B combined] *vs* 76·8% [70·9–82·0] of infants after two doses [group C]; risk difference 7·8% [90% CI 2·1–13·6]) and 23F (90·6% [95% CI 86·6–93·7] *vs* 77·6% [71·8–82·2]; 12·9% [90% CI 7·7–18·3]). Two doses at ages 2 and 6 months produced higher antibody levels than two doses at ages 2 and 4 months for all serotypes except 5 and 7F.

**Interpretation:**

A two-dose primary vaccination series was non-inferior to a three-dose primary vaccination series while two doses given with a wider interval between doses increased immunogenicity. The use of a two-dose primary vaccination schedule using a wider interval could be considered in LMIC settings to extend protection in the second year of life.

**Funding:**

Australian National Health and Medical Research Council, and The Bill & Melinda Gates Foundation.

## Introduction

Pneumococcal infections cause invasive diseases (eg, meningitis and sepsis) and mucosal diseases (eg, pneumonia and otitis media), and are a major cause of morbidity and mortality in children younger than 5 years globally.^1^ Pneumococcal conjugate vaccines (PCVs) are highly effective in preventing invasive pneumococcal disease^2^, ^3^, ^4^, ^5^ and providing indirect herd protection through reduced nasopharyngeal carriage.^6^ Two PCVs are currently in use, a ten-valent PCV (PCV10; Synflorix, GSK Vaccines, Rixensart, Belgium) containing serotypes 1, 4, 5, 6B, 7F, 9V, 14, 18C, 19F, and 23F, and a 13-valent PCV (PCV13; Prevenar, Pfizer, Philadelphia, PA, USA) that contains the ten serotypes in PCV10 plus serotypes 3, 6A, and 19A. Despite their being available for more than a decade, 43% of children globally do not have access to them because they live in countries that have yet to introduce PCV.^7^ The major barrier for access to these life-saving vaccines is their price.

**Research in context**

**Evidence before this study**

Evidence on the most appropriate and optimal pneumococcal conjugate vaccine (PCV) schedules for low-income and middle-income countries (LMICs) is lacking. Since the licensure of high-valency pneumococcal vaccines (PCVs; ten-valent PCV [PCV10] and 13-valent PCV [PCV13]), WHO has recommended either a three-dose primary series schedule in infancy without a booster dose (3 + 0) or a two-dose primary series schedule with a later booster dose (2 + 1). We searched PubMed for publications in English from database inception until Jan 31, 2020, using search terms including but not limited to “pneumococcal conjugate vaccine”, “immunogenicity” and “low- and middle-income country”. At the time this trial was designed (2012), no studies had been published on alternative PCV schedules in LMIC settings. Since then, a systematic review of 61 PCV dosing studies published in 2014 found that a three-dose primary series schedule was more immunogenic than two doses for most serotypes. Since this review has been published, three trials of different PCV schedules in LMICs have been done: two in Nepal (PCV10 in a 3 + 0 or 2 + 1 schedule, and PCV10 in a 2 + 1 schedule at ages 6 weeks, 10 weeks, and 9 months or at age 6 weeks, 14 weeks, and 9 months) and one in South Africa (PCV10 in a 3 + 1, 3 + 0, or 2 + 1 schedule). In terms of geometric mean concentration of antibodies, these trials report better responses after completion of a primary vaccination series of three doses than two doses for most serotypes, or with a dosing interval of 2 months than of 1 month for half the serotypes. Similar responses after booster vaccination were seen after vaccination with a primary series containing two or three doses, or with a 1 month or 2 month dosing interval. For many LMICs, particularly in Asia, country-specific data are needed to facilitate a decision on which vaccine schedule to introduce. Since submission of this Article, one trial in South Africa compared PCV10 and PCV13 when given as a 1 + 1 (primary dose at age 6 or 14 weeks plus booster dose at age 40 weeks) or as 2 + 1 schedule (at ages 6 and 14 weeks plus booster dose at age 40 weeks). No difference was found in the primary outcome of IgG geometric mean concentration (GMC) and GMC ratio at 1 month after booster between the single dose and two-dose primary schedules.

**Added value of this study**

To our knowledge, this is the first study to assess four different PCV10 schedules in a LMIC setting, including the WHO-recommended 3 + 0 and 2 + 1 schedules that are most likely to be implemented in high disease-burden settings, along with a 3 + 1 schedule and a novel two-dose schedule at ages 2 and 6 months. We compared the schedules using both serotype-specific IgG and opsonophagocytosis assays for all ten vaccine serotypes, and the two cross-reactive serotypes 6A and 19A to determine the potential of different PCV10 schedules to provide protective immunity to these non-vaccine types. By using both these methods, we have identified a disconnect in the IgG and opsonophagocytic assay responses for serotype 1, with adequate ELISA responses seen with inadequate opsonophagocytic assay levels after a three-dose primary series. When the third dose is given as a booster, these two responses align. This finding is of great importance in Africa, where serotype 1 is the dominant cause of pneumococcal disease and yet, with few exceptions, countries use a 3 + 0 schedule with no booster dose. We found potential benefits in increasing the between-dose interval from 2 months to 4 months. This interval could either be combined with a later booster dose or considered as a 1 + 1 schedule with an early booster dose.

**Implications of all the available evidence**

The results from this and previous studies support the immunogenicity of the WHO-recommended 2 + 1 schedule in LMIC settings. The use of a booster dose late in the first year of life should provide continued immunological protection into the second year of life for all serotypes, including serotype 1. The increased immunogenicity of a two-dose primary series schedule separated by 4 months is also promising and warrants continued investigation. Any benefits from enhanced immunogenicity would need to be balanced against a potential increased risk of disease during the interval between doses. Given the similar immunogenicity between PCV10 and PCV13 previously reported from this trial, data on the use of different PCV10 schedules are crucial for countries such as Vietnam and other LMICs in the region to make evidence-based decisions on which schedule to implement to provide optimal protection against pneumococcal disease.

PCVs were licensed on the basis of a four-dose schedule, with a three-dose primary series followed by a booster in the second year of life (3 + 1 schedule). Subsequently, three-dose schedules, either a three-dose primary series with no booster dose (3 + 0 schedule) or a two-dose primary series with a booster given at age 9 months or later (2 + 1 schedule), were shown to be effective and are recommended by WHO.^8^ A systematic review of 61 PCV dosing studies found higher serotype-specific IgG levels after a primary vaccination series with three doses than two doses for most serotypes except serotype 1, although responses were similar after the use of a booster dose for both schedules.^9^ These findings are supported by more recent studies in the Netherlands on PCV13,^10^ and in Nepal and South Africa on PCV10.^11^, ^12^ Another trial from Nepal found that a 2-month interval between doses in a primary series resulted in a higher antibody response than a 1-month interval.^13^ Therefore, timing of doses during the primary vaccination series is an important consideration because maximising protection against invasive pneumococcal disease in the intervening period until the booster dose is crucial.

Little is known of the effect of PCV dosing schedules on immunogenicity and nasopharyngeal carriage in low-income and middle-income countries (LMICs) in Asia. Therefore, we did a randomised controlled trial to determine the effects of different PCV schedules of PCV10 in infants in Vietnam, a country that has not yet introduced PCVs, and to compare vaccination responses between PCV10 and PCV13 in this setting. Immunogenicity and reactogenicity results of PCV10 and PCV13 in a 2 + 1 schedule from this trial have been reported previously.^14^ Here we report the immunogenicity findings from a range of two-dose, three-dose, and four-dose schedules of PCV10. The primary hypothesis is that a two-dose primary series of PCV10 is non-inferior (at the 10% level) to a three-dose primary series, in terms of the proportion of participants with serotype-specific IgG concentrations of 0·35 μg/mL or higher.

## Methods

### Study design and participants

Detailed methods for this single-blind, parallel-group, open-label, randomised, controlled trial have been published previously.^15^ Briefly, infants with no clinically significant maternal or perinatal history and who were born at or after 36 weeks' gestation were recruited at age 2 months by community health staff in districts 4 and 7 in Ho Chi Minh City, Vietnam, and were followed-up until age 24 months. Infants were excluded if they had any known allergy to any component of the vaccine or previously had an allergic or anaphylactic reaction to any vaccine, had a known immunodeficiency disorder, or were born to a mother with HIV. Full details of the participant eligibility criteria and recruitment processes have been described previously.^15^

Parents or legal guardians provided written informed consent for each participant. The study was approved by the Human Research Ethics Committee of the Northern Territory Department of Health and Menzies School of Health Research (EC00153) and the Vietnam Ministry of Health Ethics Committee. The protocol was also approved by the Institutional Review Board at the Pasteur Institute of Ho Chi Minh City. The protocol has been published previously.^15^

### Randomisation and masking

Participants were randomly assigned (3:3:5:4:5:4), using a computer-generated list of randomisation numbers and a block randomisation scheme,^15^ stratified by district, to one of six groups (A–F). Participants in groups A–D were assigned to vaccination with PCV10 in one of four schedules: four doses at ages 2, 3, 4, and 9 months in a 3 + 1 schedule (group A); three doses at ages 2, 3, and 4 months in a 3 + 0 schedule (group B); three doses at ages 2, 4, and 9·5 months in a 2 + 1 schedule (group C); or two doses at ages 2 and 6 months in a two-dose schedule (group D). Groups A–D contribute data to this analysis. Groups E and F are not included because participants in group E were given PCV13 and participants in group F did not receive any infant doses of PCV.^15^ All laboratory-based outcome assessors were masked to the group allocation, because the key outcome measures that address the study objectives are all laboratory-based. The parents or guardians of participants and clinic staff administering the vaccinations were unmasked to group allocation because of the nature of the intervention. Additional details of the randomisation and masking procedures have been described previously.^15^

### Procedures

In addition to the PCV10 vaccine according to their allocated schedules, participants also received four doses of the hexavalent diphtheria, tetanus, pertussis, polio, *Haemophilus influenzae* type b, and hepatitis B (DTaP-IPV-Hib-HepB) vaccine and two doses of measles-containing vaccines (appendix p 2). In group C, the booster dose of PCV10 (and DTaP-IPV-Hib-HepB) was administered at 9·5 months because the Vietnamese Ministry of Health does not permit co-administration of measles (first dose at 9 months) and DTaP-IPV-Hib-HepB. Vaccination visits were scheduled within specified time windows, defined either by time since the previous dose of vaccine or by age of the participant (appendix p 2).

A maximum of four blood samples per participant during the study period was permitted by the Vietnamese Ministry of Health. Therefore, samples at some timepoints were only collected from a subset of participants. Blood samples were collected after one dose of PCV10 (at age 3 months in group D), after two doses of PCV10 (at age 5 months in group C and at age 7 months in group D), and after three doses of PCV10 (at age 5 months in groups A and B). Blood samples were also collected at age 6 months (groups B and D, and a subset of group C), 9 months (groups A and C, and a subset of groups B and D), 10 months (groups A, B, and C), and 18 months (a subset of groups A–D; appendix p 2). Blood samples taken after each dose of PCV10 had a collection window of 27–43 days after vaccination.

Serotype-specific IgG concentrations to all serotypes in PCV10 and the two cross-reactive serotypes (6A, 19A) were measured using a modified WHO ELISA method that has been described previously.^16^ Opsonophagocytic assays were done on the basis of a previously published multiplexed opsonophagocytic assay method^17^ to all PCV10 serotypes and the cross-reactive serotypes 6A and 19A.

### Outcomes

The primary outcome measure was the proportion of infants with protective levels of antibodies, defined as IgG concentration of 0·35 μg/mL or higher, measured by ELISA. The primary outcome timepoint was 4 weeks after the primary vaccination series, comparing three doses of PCV10 given at age 2, 3, and 4 months (groups A and B combined) with two doses given at age 2 and 4 months (group C). We also assessed the dosing interval in a two-dose primary series, comparing doses given at ages 2 and 4 months (group C, assessed at age 5 months) and at ages 2 and 6 months (group D, assessed at age 7 months). Additional timepoints were before and after the booster dose, and at age 18 months.

Secondary outcome measures were geometric mean concentrations (GMCs) of serotype-specific IgG antibodies, and functional antibody responses assessed with opsonophagocytic assay in terms of the proportion of participants with an opsonisation index (OI) of at least 8 and geometric mean opsonisation indices (GMOIs). Opsonophagocytic assays were done 4 weeks after the primary vaccination series (groups A and B combined, C, and D) and 4 weeks after the booster vaccination (groups A, B, and C) in half of the participants; for logistical reasons these participants were the first half of each group with samples collected at both timepoints. Data on serious adverse events were collected throughout the study, through parent-report and regular review of hospital records.

### Statistical analysis

We present primary comparisons between groups in terms of the proportion of participants with a serotype-specific IgG concentration of at least 0·35 μg/mL at 4 weeks after finishing their allocated primary vaccination series. We calculated risk differences with Newcombe-Score CIs, with a 10% difference considered to be meaningful, in line with the pivotal studies that were used to support the licensure of PCV10 and PCV13.^18^, ^19^, ^20^ The two-dose and the three-dose primary vaccination series were compared in terms of non-inferiority. The null hypothesis for each of the vaccine serotypes was that the risk difference was greater than 10%, and was rejected if the upper bound of the 90% CI was less than 10% (equivalent to using a 5% one-sided test). Details of the sample size calculations have been described previously.^15^ The sample size provided more than 99% power for an overall conclusion of non-inferiority, drawn if the null hypothesis was rejected for at least seven of the ten vaccine serotypes. For the comparison of two doses administered at ages 2 and 4 months and at ages 2 and 6 months, the null hypothesis for each of the vaccine serotypes was that the risk difference was between −10% and 10%, with the null hypothesis rejected if the 95% CI of the risk difference was entirely outside of this range. An overall difference was declared if at least seven of the ten individual null hypotheses were rejected in the same direction.

We also compared serotype-specific IgG concentrations between groups in terms of the GMC ratios between the three-dose and two-dose primary series groups (group A and B combined *vs* group C) and the two two-dose primary vaccination series groups (group D *vs* group C) with 95% CIs, and were described as higher in one group than another if the 95% CI did not cross 1·00. Similarly, GMOIs were described as higher in one group than another if the 95% CI of the GMOI ratio excluded 1·00. We calculated risk differences for the proportions of participants with an OI of at least 8, and the risk difference was determined to be higher in one group than another if the 95% CI excluded zero.

Beyond the primary outcome, we aimed to provide an overview of any patterns of differences in immunogenicity between the schedules. We did not make any formal adjustments for multiple comparisons, but we deliberately avoided reporting p values.

We did all immunological analyses on the per-protocol population (ie, all participants with post-vaccination blood samples collected within the specified window) and analyses of the primary endpoint were repeated in the intention-to-treat population (including all available blood samples). A post-hoc analysis was done comparing the immunogenicity after completion of the two-dose schedule and the 2+1 schedule.

We did all analyses using Stata Statistical Software (release 14). This study is registered with ClinicalTrials.gov, NCT01953510.

### Role of the funding source

The funders of the study had no role in study design, data collection, data analysis, data interpretation, or writing of the report.

## Results

**Figure 1:**
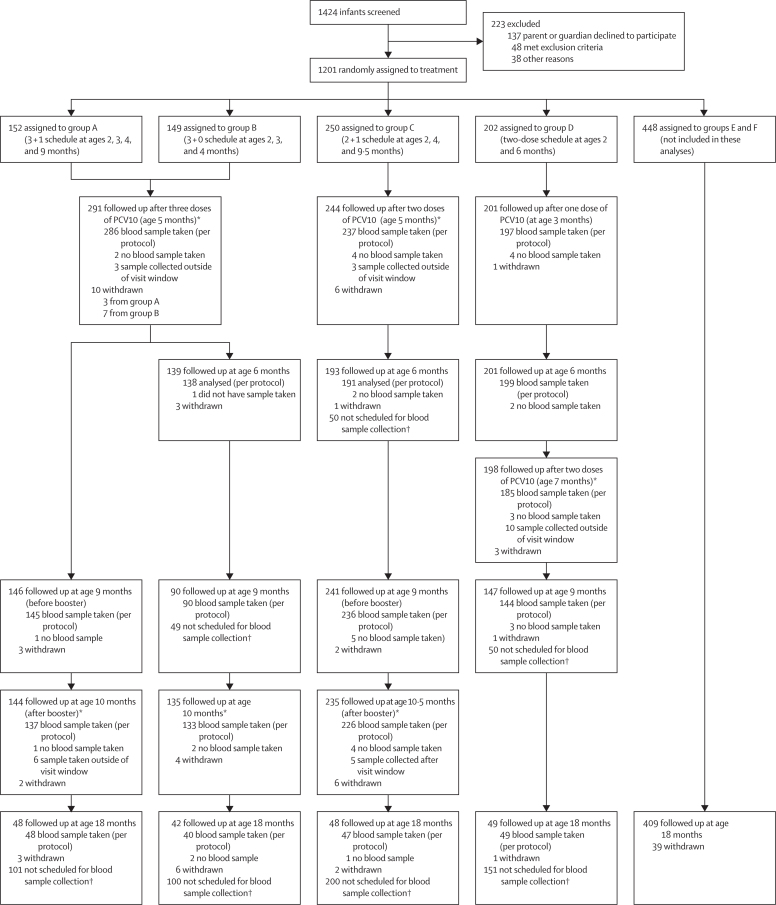
Trial profile Samples collected outside the visit window (27–43 days after vaccination) were included only in the intention-to-treat analyses. Full details of the trial have been described previously.^15^ *Half of participants in groups A–D contributed to the opsonophagocytic assay analyses. †Participants allocated to groups A–D from the last 300 recruited provided a blood sample at age 18 months, with the remainder providing a sample at an alternative timepoint, because a maximum of four blood samples was permitted per participant over the course of the study.

Between Sept 30, 2013, and Jan 9, 2015, 1424 infants were referred from community health centres for screening and 1201 were enrolled and randomly assigned to groups A–F (figure 1). 1093 (91%) of 1201 participants were followed up until age 18 months. As previously reported,^14^ groups A–D were balanced with respect to baseline characteristics (appendix p 3). 388 (52%) of 753 participants in groups A–D were female and 365 (48%) were male. Among participants in groups A–D contributing to these analyses, 708 (94%) of 753 completed their primary vaccination series and had blood draws within the specified time window (286 [95%] of 301 in groups A and B, 237 [95%] of 250 in group C, and 185 [92%] of 202 in group D; figure 1). Of 2172 doses of PCV10, 2126 (97·9%) were administered within the specified time window. Of 1295 blood samples taken, 1268 (97·9%) were collected within the required window, with the other 27 samples excluded from analyses.

After the final dose of the primary vaccination series, two doses at ages 2 and 4 months (group C) were non-inferior to three doses at ages 2, 3, and 4 months (groups A and B combined) in terms of the proportion of infants with protective serotype-specific IgG concentrations of 0·35 μg/mL or higher for eight of ten vaccine serotypes, thereby meeting the criteria for the overall conclusion of non-inferiority of at least seven out of ten serotypes (table 1; appendix p 4). The proportion of participants with protective levels was higher in the combined three-dose group than in the two-dose group for serotype 6B and serotype 23F (table 1), and the proportions with protective levels for the other eight serotypes were more than 95% in both groups. Comparing GMCs, serotype-specific IgG concentrations were higher in the combined three-dose group than in the two-dose group for all PCV10 serotypes except 19F, for which the 95% CI of the GMC ratio crossed 1·00 (table 1). For the cross-reactive serotypes 6A and 19A, the proportion of participants with protective levels and the IgG GMC were higher in the combined three-dose group than in the two-dose group for serotype 6A but not for 19A (table 1). The per-protocol and intention-to-treat analyses gave similar results (appendix p 5).

**Table 1:** Immunogenicity of PCV10 after the primary vaccination series in the per-protocol population

	**Proportion of participants with IgG concentration ≥0·35 μg/mL**			**Risk difference (primary outcome)**		**GMC, μg/mL**			**GMC ratio**	
	Groups A and B combined (n=286)	Group C (n=237)	Group D (n=185)	Groups A and B combined *vs* group C (90% CI)	Group D *vs* group C (95% CI)	Groups A and B combined (n=286)	Group C (n=237)	Group D (n=185)	Groups A and B combined *vs* group C	Group D *vs* group C
**Vaccine serotypes**										
1	98·3% (96·0 to 99·4)	97·9% (95·1 to 99·3)	99·5% (97·0 to 100)	0·4% (−1·7 to 2·7)	1·6% (−1·2 to 4·3)	2·79 (2·51 to 3·10)	2·21 (1·97 to 2·48)	3·40 (3·01 to 3·84)	1·26 (1·08 to 1·47)	1·54 (1·30 to 1·82)
4	99·0% (97·0 to 99·8)	98·7% (96·3 to 99·7)	100% (98·0 to 100)	0·2% (−1·5 to 2·2)	1·3% (−0·9 to 3·7)	3·85 (3·44 to 4·31)	3·21 (2·87 to 3·58)	4·28 (3·83 to 4·79)	1·20 (1·03 to 1·41)	1·34 (1·14 to 1·57)
5	98·6% (96·5 to 99·6)	95·8% (92·4 to 98·0)	98·4% (95·3 to 99·7)	2·8% (0·4 to 5·6)	2·6% (−1·0 to 6·1)	1·81 (1·67 to 1·97)	1·17 (1·07 to 1·27)	1·31 (1·19 to 1·43)	1·55 (1·38 to 1·75)	1·12 (0·99 to 1·26)
6B	84·6% (79·9 to 88·6)	76·8% (70·9 to 82·0)	96·2% (92·4 to 98·5)	7·8% (2·1 to 13·6)	19·4% (13·2 to 25·5)	1·08 (0·95 to 1·23)	0·80 (0·69 to 0·92)	2·59 (2·25 to 2·99)	1·36 (1·12 to 1·64)	3·25 (2·66 to 3·97)
7F	99·3% (97·5 to 99·9)	98·7% (96·3 to 99·7)	100% (98·0 to 100)	0·6% (−1·0 to 2·5)	1·3% (−0·9 to 3·7)	3·04 (2·79 to 3·32)	2·07 (1·89 to 2·27)	2·20 (2·01 to 2·40)	1·47 (1·29 to 1·66)	1·06 (0·93 to 1·21)
9V	99·3% (97·5 to 99·9)	96·2% (92·9 to 98·2)	98·9% (96·1 to 99·9)	3·1% (1·0 to 5·8)	2·7% (−0·6 to 6·1)	2·47 (2·26 to 2·71)	1·63 (1·47 to 1·81)	2·40 (2·17 to 2·66)	1·51 (1·32 to 1·74)	1·47 (1·27 to 1·71)
14	100% (98·7 to 100)	98·3% (95·7 to 99·5)	98·9% (96·1 to 99·9)	1·7% (0·4 to 3·7)	0·6% (−2·4 to 3·3)	9·76 (8·79 to 10·83)	5·86 (5·11 to 6·73)	8·59 (7·43 to 9·94)	1·66 (1·41 to 1·97)	1·47 (1·20 to 1·79)
18C	98·6% (96·5 to 99·6)	96·6% (93·5 to 98·5)	98·4% (95·3 to 99·7)	2·0% (−0·3 to 4·6)	1·8% (−1·7 to 5·1)	3·87 (3·47 to 4·30)	1·86 (1·64 to 2·11)	2·95 (2·61 to 3·34)	2·08 (1·76 to 2·44)	1·59 (1·33 to 1·89)
19F	99·7% (98·1 to 100)	99·2% (97·0 to 99·9)	100% (98·0 to 100)	0·5% (−0·8 to 2·2)	0·8% (−1·3 to 3·0)	8·34 (7·52 to 9·24)	9·54 (8·37 to 10·87)	16·47 (14·50 to 18·72)	0·87 (0·74 to 1·03)	1·73 (1·43 to 2·08)
23F	90·6% (86·6 to 93·7)	77·6% (71·8 to 82·8)	93·5% (88·9 to 96·6)	12·9% (7·7 to 18·3)	15·9% (9·3 to 22·2)	1·32 (1·18 to 1·48)	0·89 (0·78 to 1·02)	2·17 (1·90 to 2·48)	1·48 (1·24 to 1·77)	2·43 (2·00 to 2·95)
**Cross-reacting serotypes**										
6A	50·3% (44·4 to 56·3)	40·5% (34·2 to 47·1)	69·2% (62·0 to 75·8)	9·8% (2·6 to 16·9)	28·7% (19·2 to 37·4)	0·37 (0·34 to 0·41)	0·31 (0·28 to 0·35)	0·64 (0·55 to 0·73)	1·19 (1·03 to 1·37)	2·04 (1·71 to 2·43)
19A	68·2% (62·4 to 73·5)	70·5% (64·2 to 76·2)	85·9% (80·1 to 90·6)	−2·3% (−8·9 to 4·4)	15·5% (7·6 to 22·9)	0·56 (0·51 to 0·62)	0·55 (0·49 to 0·62)	0·93 (0·81 to 1·06)	1·02 (0·88 to 1·19)	1·68 (1·41 to 2·01)

Data in parentheses are 95% CI, unless otherwise specified. Immunogenicity data are for 4 weeks after completion of the primary vaccination series. In their primary vaccination series, group A and B were both given three doses at ages 2, 3, and 4 months and their data are combined for the purpose of this analysis, group C were given two doses at ages 2 and 4 months, and group D were given two doses at ages 2 and 6 months. GMC=geometric mean concentration. PCV10=ten-valent pneumococcal conjugate vaccine.

Analysis of functional antibody levels after the primary vaccination series by opsonophagocytosis revealed higher GMOIs (with the 95% CI of the GMOI ratio entirely >1·00) after three doses at ages 2, 3, and 4 months (groups A and B combined) than two doses at ages 2 and 4 months (group C) for eight vaccine serotypes (all except 4 and 19F) and the cross-reactive serotype 6A (table 2). For six serotypes (1, 9V, 14, 18C, 23F, and 6A), a higher proportion of participants in the combined three-dose group than in the two-dose group had an OI of 8 or higher (table 2).

**Table 2:** Functional antibody responses to PCV10 in the per-protocol population after the primary vaccination series

	**Proportion of participants with an opsonisation index of ≥8**			**Risk difference**		**Geometric mean opsonisation index**			**Ratio of geometric mean opsonisation indices**	
	Groups A and B combined (n=147)*	Group C (n=124)*	Group D (n=97)*	Groups A and B combined *vs* group C	Group D *vs* group C	Groups A and B combined (n=147)*	Group C (n=124)*	Group D (n=97)*	Groups A and B combined *vs* group C	Group D *vs* group C
**Vaccine serotypes**										
1	77·6% (69·9 to 84·0)	66·1% (57·1 to 74·4)	87·6% (79·4 to 93·4)	11·4% (0·7 to 22·0)	21·5% (10·4 to 31·6)	37 (29 to 47)	22 (17 to 28)	85 (62 to 118)	1·67 (1·17 to 2·39)	3·86 (2·58 to 5·79)
4	99·3% (96·3 to 100)	100% (97·1 to 100)	100% (96·3 to 100)	−0·7% (−3·8 to 2·4)	0·0% (−3·8 to 3·0)	889 (766 to 1032)	922 (820 to 1036)	1056 (912 to 1223)	0·96 (0·79 to 1·17)	1·15 (0·95 to 1·38)
5	100% (97·5 to 100)	97·6% (93·1 to 99·5)	96·9% (91·2 to 99·4)	2·4% (−0·6 to 6·9)	−0·7% (−6·5 to 4·2)	461 (390 to 546)	351 (286 to 430)	582 (453 to 749)	1·32 (1·01 to 1·71)	1·66 (1·21 to 2·28)
6B	81·6% (74·4 to 87·5)	71·8% (63·0 to 79·5)	90·7% (83·1 to 95·7)	9·9% (−0·2 to 19·9)	18·9% (8·6 to 28·5)	112 (78 to 162)	59 (40 to 86)	160 (111 to 233)	1·90 (1·12 to 3·22)	2·71 (1·58 to 4·65)
7F	97·3% (93·2 to 99·3)	96·8% (91·9 to 99·1)	96·9% (91·2 to 99·4)	0·5% (−4·0 to 5·6)	0·1% (−5·8 to 5·3)	511 (390 to 669)	250 (182 to 343)	466 (348 to 623)	2·04 (1·35 to 3·09)	1·86 (1·20 to 2·90)
9V	93·9% (88·7 to 97·2)	80·6% (72·6 to 87·2)	99·0% (94·4 to 100)	13·2% (5·4 to 21·6)	18·3% (10·8 to 26·2)	163 (124 to 213)	73 (52 to 102)	253 (186 to 343)	2·22 (1·45 to 3·40)	3·46 (2·18 to 5·50)
14	98·0% (94·2 to 99·6)	89·5% (82·7 to 94·3)	94·8% (88·4 to 98·3)	8·4% (2·7 to 15·2)	5·3% (−2·3 to 12·6)	460 (348 to 609)	132 (92 to 191)	296 (210 to 417)	3·48 (2·21 to 5·47)	2·24 (1·34 to 3·74)
18C	96·6% (92·2 to 98·9)	88·7% (81·8 to 93·7)	95·9% (89·8 to 98·9)	7·9% (1·7 to 14·9)	7·2% (−0·3 to 14·4)	361 (282 to 463)	124 (88 to 175)	329 (239 to 453)	2·91 (1·93 to 4·39)	2·65 (1·65 to 4·26)
19F	98·6% (95·2 to 99·8)	100% (97·1 to 100)	99·0% (94·4 to 100)	−1·4% (−4·8 to 1·8)	−1·0% (−5·6 to 2·1)	1263 (1078 to 1481)	1217 (1078 to 1375)	1133 (936 to 1371)	1·04 (0·85 to 1·27)	0·93 (0·75 to 1·16)
23F	80·3% (72·9 to 86·4)	58·9% (49·7 to 67·6)	84·5% (75·8 to 91·1)	21·4% (10·5 to 31·8)	25·7% (13·8 to 36·2)	69 (50 to 94)	29 (21 to 41)	74 (53 to 104)	2·36 (1·48 to 3·76)	2·55 (1·56 to 4·17)
**Cross-reacting serotypes**										
6A	46·9% (38·7 to 55·3)	31·5% (23·4 to 40·4)	60·8% (50·4 to 70·6)	15·5% (3·8 to 26·5)	29·4% (16·2 to 41·2)	40 (26 to 61)	18 (12 to 26)	77 (46 to 130)	2·29 (1·27 to 4·11)	4·39 (2·31 to 8·33)
19A	35·4% (27·7 to 43·7)	35·5% (27·1 to 44·6)	44·3% (34·2 to 54·8)	−0·1% (−11·5 to 11·1)	8·8% (−4·1 to 21·5)	9 (8 to 12)	9 (7 to 11)	16 (11 to 23)	1·09 (0·80 to 1·49)	1·90 (1·29 to 2·81)

Data in parentheses are 95% CIs Functional antibody responses after the primary vaccination series in groups A and B combined (three doses at ages 2, 3, and 4 months), group C (two doses at ages 2 and 4 months), and group D (two doses at ages 2 and 6 months).

* Opsonophagocytic assays were done in half of participants per group (150 from groups A and B combined, 125 from group C, and 100 from group D); however, seven participants (three from group B, one from group C, and three from group D) had their blood sample collected outside of the study window (23–43 days after vaccination) so were excluded from the analysis.

Comparing two doses administered at ages 2 and 4 months (group C) with two doses administered at ages 2 and 6 months (group D), only serotype 6B showed a difference in the proportion of participants with protective IgG levels at the 10% level, being higher with vaccination at ages 2 and 6 months than at ages 2 and 4 months (table 1; appendix p 4). IgG GMCs were higher with vaccination at ages 2 and 6 month group than at ages 2 and 4 months for all vaccine serotypes except 5 and 7F (table 1; appendix pp 6). For four of ten vaccine serotypes (1, 6B, 19F, and 23F), IgG GMCs were higher after two-dose vaccination at ages 2 and 6 months than after the three-dose primary series (data not shown). Among the cross-reactive serotypes, the proportion of infants with IgG concentrations of 0·35 μg/mL or higher was greater with two-dose vaccination at ages 2 and 6 months than at ages 2 and 4 months for serotype 6A. The IgG GMCs were higher with two-dose vaccination at ages 2 and 6 months than at ages 2 and 4 months for both serotype 6A and 19A (table 1).

In relation to functional antibody responses, higher GMOIs were seen with vaccination at ages 2 and 6 months than at ages 2 and 4 months for eight vaccine serotypes (all except 4 and 19F), and the cross-reactive serotype 6A (table 2; appendix p 6). A higher proportion of participants vaccinated at ages 2 and 6 months than at ages 2 and 4 months had an OI of 8 or higher for serotypes 1, 6B, 9V, 23F, and 6A (table 2).

**Figure 2:**
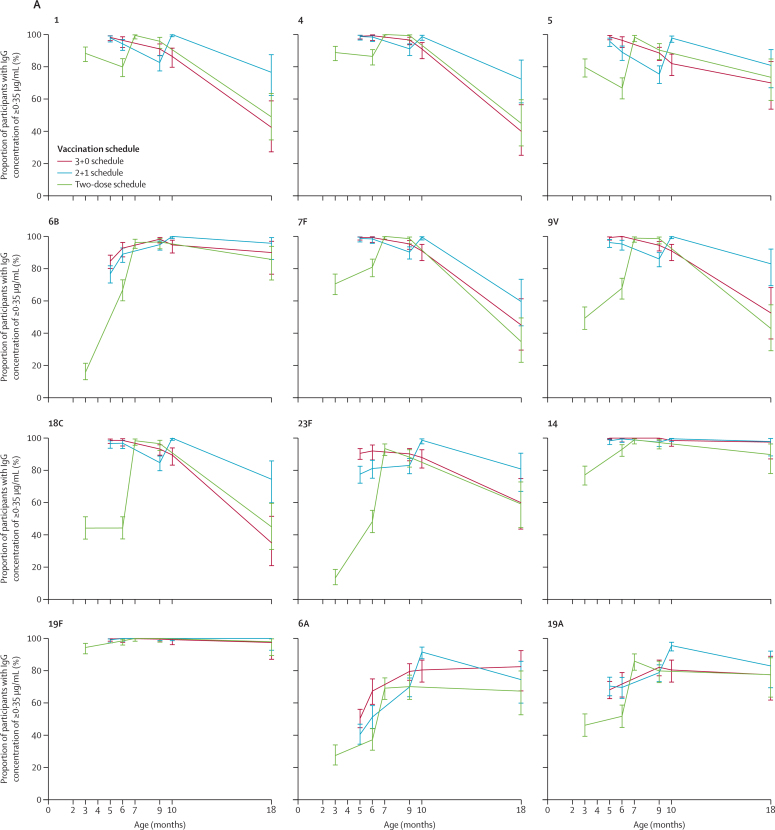

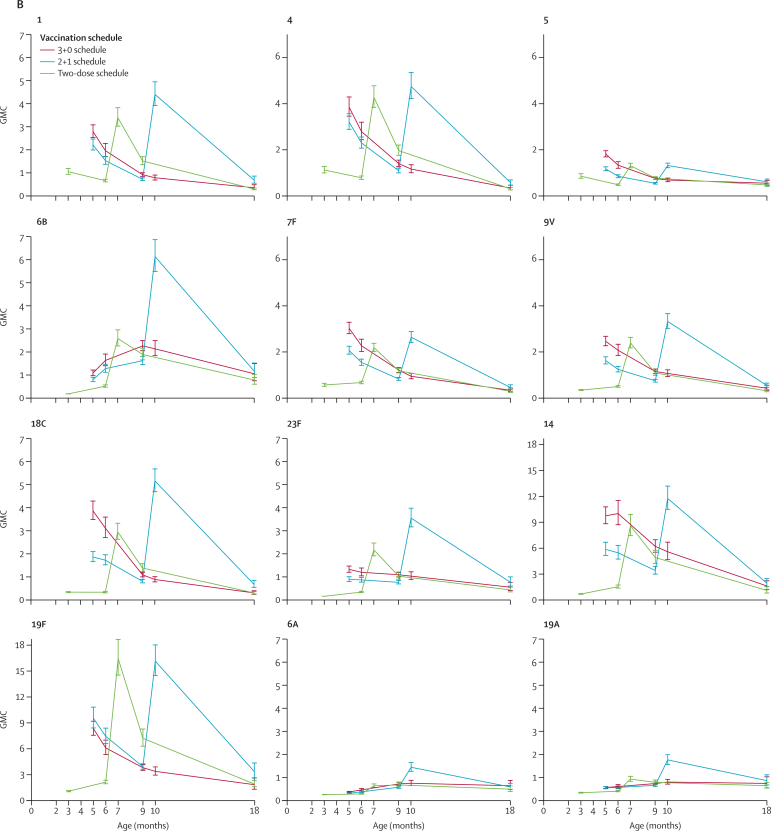
Proportion of participants with serotype-specific IgG concentrations ≥0·35 μg/mL over time (A) and geometric mean serotype-specific IgG concentrations (B), for all PCV10 serotypes as well as the two cross-reacting serotypes Datapoints are percentage or GMC with error bars showing 95% CIs. Data for the 3+0 schedule was sourced from groups A and B combined (ages 5 months and 9 months) or group B. Data for the 2+1 schedule was sourced from group C and for the two-dose schedule was sourced from group D. The y-axis range is longer for serotypes 14 and 19F because these serotypes were more immunogenic. GMC=geometric mean concentration. PCV10=ten-valent pneumococcal conjugate vaccine.

We assessed the immunogenicity of a 3 + 0 schedule (group B), 2 + 1 schedule (group C), and two-dose schedule (group D) longitudinally, using all available blood samples (figure 2; appendix pp 7–8). All three schedules were immunogenic in terms of the proportion of participants with antibody concentrations of 0·35 μg/mL or higher, with over three-quarters of participants reaching this concentration after their primary vaccination series through to age 9 or 10 months for each of the vaccine serotypes (figure 2). Over 70% of participants also had antibody concentrations of 0·35 μg/mL or higher after a single dose of PCV10 at age 2 months for six serotypes (1, 4, 5, 7F, 14, and 19F; figure 2); although, as reported previously,^14^ the proportion of participants who had this concentration of antibodies for serotype 14 did not increase significantly after vaccination compared with before vaccination. By age 18 months, the proportion of participants with antibody concentrations of 0·35 μg/mL or higher had decreased for most serotypes, ranging 59·6–100·0% for the 2 + 1 schedule (group C), 35·0–97·5% for the 3 + 0 schedule (group B), and 34·7–98·0% for the two-dose group (group D; figure 2; appendix p 8).

Assessing the GMCs longitudinally, a similar profile for most PCV10 serotypes was seen comparing the two-dose and 2 + 1 schedules, with peak IgG GMCs observed after the respective final doses at age 6 months for the two-dose schedule (group D) and 9·5 months for the 2 + 1 schedule (group C; figure 2). These peak IgG GMCs were higher with the 2 + 1 schedule than with the two-dose schedule for seven of the PCV10 serotypes (1, 6B, 7F, 9V, 14, 18C, and 23F; figure 2, appendix p 6). Higher GMCs were also seen with the 2 + 1 schedule than the two-dose schedule at age 18 months for all PCV10 serotypes except 5 and 6B (appendix p 8). Comparing the 3 + 0 and 2 + 1 schedules, the pattern of differences seen after completion of the primary vaccination series continued until age 9 months, with GMCs at both ages 6 and 9 months being higher with the 3 + 0 schedule than with the 2 + 1 schedule for all vaccine serotypes except 19F (figure 2). At age 10 months, GMCs were higher with the 2 + 1 schedule than with the 3 + 0 schedule for all serotypes, and also at age 18 months for four serotypes (1, 4, 18C, and 19F).

For the cross-reactive serotypes 6A and 19A, we found a substantial response at age 9 or 10 months for each of the schedules, with the proportion of participants with antibody concentrations of 0·35 μg/mL or higher for both serotypes being over 91% with the 2 + 1 schedule (group C; at 10 months), over 79% with the 3 + 0 schedule (group B; at both 9 and 10 months), and over 70% with the two-dose schedule (group D; at 9 months; figure 2, appendix p 7). By age 18 months, these proportions were still over 74% with the 2 + 1 schedule, over 77% with the 3 + 0 schedule, and over 67% with the two-dose schedule (figure 2; appendix p 8). We found no differences between the schedules at age 18 months for these cross-reactive serotypes with regards to both the proportion of participants with antibody concentrations of 0·35 μg/mL or higher and the IgG GMCs (figure 2; appendix p 8).

We also compared the responses before and after booster vaccinations for the 3 + 1 (group A) and 2 + 1 (group C) schedules. Before booster, most participants had IgG concentrations of 0·35 μg/mL or higher for each serotype (>89% in group A and >75% in group C), but a greater proportion was seen with the 3 + 1 schedule than with the 2 + 1 schedule for five serotypes (1, 4, 5, 9V, and 18C; appendix p 7). IgG GMCs were higher with the 3 + 1 schedule than with the 2 + 1 schedule for nine PCV10 serotypes (all except 19F). After booster vaccination, we found no difference in the proportion of participants with IgG concentrations of 0·35 μg/mL or higher between the 3 + 1 and 2 + 1 schedules, with more than 97% of participants meeting this level in both groups and across all serotypes. However, IgG GMCs were higher with the 3 + 1 schedule than with the 2 + 1 schedule for eight PCV10 serotypes (all except 6B and 19F; appendix p 7). For the cross-reactive serotypes 6A and 19A, we found no differences between the 3 + 1 and 2 + 1 schedules in terms of the proportion of participants who had IgG concentrations of 0·35 μg/mL or higher or the IgG GMCs, either before or after booster vaccination (appendix p 7).

We also compared the 3 + 1 and 2 + 1 schedules after booster vaccination in terms of functional antibody levels. After booster vaccination, more than 97% of participants given the 3 + 1 schedule (group A) and more than 90% given the 2 + 1 schedule (group C) had an OI of 8 or higher across the ten vaccine serotypes, with no differences between the schedules (table 3). Higher GMOIs were seen with the 3 + 1 schedule than with the 2 + 1 schedule for five PCV10 serotypes (4, 5, 7F, 14, and 23F; table 3). Among participants who were given the 3 + 0 schedule (group B), more than 73% had an OI of 8 or higher at age 10 months to all PCV10 serotypes except serotype 1, although the GMOIs were notably lower in this group than those in both the booster-containing schedules (table 3).

**Table 3:** Functional antibody responses to PCV10 in the per-protocol population 4 weeks after booster vaccination (or at age 10 months for group B)

	**Proportion of participants with an opsonisation index of ≥8**			**Risk difference between group A and group C**	**Geometric mean opsonisation index**			**Ratio of geometric mean opsonisation indices between group A and group C**
	Group A (3+1 schedule; n=71)*	Group C (2+1 schedule; n=121)*	Group B (3+0 schedule; n=75)†		Group A (3+1 schedule; n=71)*	Group B (2+1 schedule; n=121)*	Group C (3+0 schedule; n=75)†	
**Vaccine serotypes**								
1	97·2% (90·2 to 99·7)	90·9% (84·3 to 95·4)	42·7% (31·3 to 54·6)	6·3% (−1·7 to 13·0)	191 (135 to 270)	145 (106 to 198)	10 (8 to 14)	1·32 (0·81 to 2·14)
4	100% (94·9 to 100)	99·2% (95·5 to 100)	85·1% (75·0 to 92·3)	0·8% (−4·4 to 4·5)	1836 (1463 to 2304)	1280 (1072 to 1529)	152 (96 to 239)	1·43 (1·08 to 1·91)
5	100% (94·9 to 100)	98·3% (94·2 to 99·8)	95·9% (88·6 to 99·2)	1·7% (−3·6 to 5·8)	1145 (913 to 1436)	768 (627 to 941)	103 (75 to 142)	1·49 (1·09 to 2·04)
6B	100% (94·9 to 100)	96·7% (91·8 to 99·1)	81·3% (70·7 to 89·4)	3·3% (−2·2 to 8·2)	425 (306 to 590)	299 (224 to 399)	73 (46 to 114)	1·42 (0·91 to 2·23)
7F	100% (94·9 to 100)	100% (97·0 to 100)	93·3% (85·1 to 97·8)	0·0% (−5·1 to 3·1)	913 (639 to 1306)	484 (369 to 636)	165 (113 to 242)	1·89 (1·21 to 2·95)
9V	100% (94·9 to 100)	94·2% (88·4 to 97·6)	88·0% (78·4 to 94·4)	5·8% (−0·1 to 11·5)	483 (332 to 702)	308 (217 to 436)	74 (50 to 110)	1·57 (0·92 to 2·68)
14	100% (94·9 to 100)	96·7% (91·8 to 99·1)	90·7% (81·7 to 96·2)	3·3% (−2·2 to 8·2)	754 (548 to 1038)	394 (293 to 531)	134 (88 to 205)	1·91 (1·21 to 3·02)
18C	100% (94·9 to 100)	99·2% (95·5 to 100)	76·0% (64·7 to 85·1)	0·8% (−4·4 to 4·5)	1039 (779 to 1387)	732 (564 to 950)	35 (24 to 51)	1·42 (0·95 to 2·12)
19F	100% (94·9 to 100)	100% (97·0 to 100)	94·6% (86·7 to 98·5)	0·0% (−5·1 to 3·1)	1807 (1431 to 2281)	1579 (1380 to 1807)	186 (135 to 256)	1·14 (0·89 to 1·47)
23F	97·2% (90·2 to 99·7)	91·7% (85·3 to 96·0)	73·3% (61·9 to 82·9)	5·4% (−2·4 to 12·1)	262 (181 to 378)	149 (109 to 202)	35 (24 to 52)	1·76 (1·08 to 2·86)
**Cross-reacting serotypes**								
6A	78·9% (67·6 to 87·7)	66·9% (57·8 to 75·2)	48·6% (36·9 to 60·6)	11·9% (−1·4 to 23·7)	211 (122 to 364)	118 (74 to 189)	38 (21 to 68)	1·79 (0·85 to 3·74)
19A	69·0% (56·9 to 79·5)	61·2% (51·9 to 69·9)	18·7% (10·6 to 29·3)	7·9% (−6·3 to 20·9)	38 (24 to 59)	25 (18 to 34)	6 (5 to 7)	1·52 (0·89 to 2·61)

Data in parentheses are 95% CIs. Serotype-specific geometric mean opsonisation index and percentage of participants with an opsonisation index of ≥8 at 4 weeks after the booster vaccination for group A (3+1 schedule at ages 2, 3, 4, and 9 months); group C (2+1 schedule at ages 2, 4, and 9·5 months); and group B (3+0 schedule at ages 2, 3, and 4 months).

* Opsonophagocytic assays were done in half of participants per group (75 from group A, 125 from group C, and 75 from group B); however, eight participants (four from group A and four from group C) had their post-booster blood sample collected outside of the study window (23–43 days after vaccination) and so were excluded from the analysis.

† n=74 for serotypes 4, 5, 19F, and 6A because one sample in group B was not tested because insufficient serum sample was collected.

For most serotypes, the opsonophagocytic assay results mirrored the IgG data, as seen with serotypes 18C and 6B (appendix pp 9). For some serotypes, most notably serotypes 1 and 23F, a relatively low proportion of participants had OIs of 8 or higher after completion of the primary vaccination series compared with the proportion who had IgG concentrations of 0·35 μg/mL or higher, especially those given the 2 + 1 schedule (group C; 66·1% had an OI of ≥8 *vs* 97·9% had an IgG concentration of ≥0·35 μg/mL for serotype 1, and 58·9% had an OI of ≥8 *vs* 77·6% had an IgG concentration of ≥0·35 μg/mL for serotype 23F; appendix p 9). After booster vaccination, more than 90% of participants given the 2 + 1 schedule had an OI of 8 or higher for both these serotypes, and 100% had an IgG concentration of 0·35 μg/mL or higher for serotype 1 and 98·7% had IgG concentration of 0·35 μg/mL or higher for serotype 23F (appendix p 9).

Because of the favourable immunogenicity of the two-dose schedule compared with the 2 + 1 schedule after completion of the primary vaccination series, we did a further post-hoc comparison at age 7 months in group D and at age 10 months in group C (appendix pp 6, 10). The IgG concentrations at age 10 months after a 2 + 1 schedule were higher than those at age 7 months after a two-dose schedule for seven PCV10 serotypes (figure 2, appendix p 6). The exceptions were serotypes 4, 5 and 19F, with GMCs of 4·75 μg/mL (95% CI 4·20–5·37) for serotype 4, 1·31 μg/mL (1·20–1·43) for serotype 5, and 16·16 μg/mL (14·45–18·08) for serotype 19F with the 2 + 1 schedule compared with 4·28 μg/mL (3·83–4·79) for serotype 4, 1·31 μg/mL (1·19–1·43) for serotype 5, and 16·47 μg/mL (14·50–18·72) for serotype 19F with the two-dose schedule. In terms of functional antibody levels, the 2 + 1 schedule mostly resulted in higher GMOIs than the two-dose schedule for five PCV10 serotypes; the exceptions were serotypes 4, 5, 7F, 9V, and 14 (appendix pp 6, 10).

## Discussion

WHO recommends the use of PCV10 or PCV13 in either a 3 + 0 or 2 + 1 schedule,^8^ leaving countries with decisions on which vaccine to choose and in what schedule. We set out to identify the most efficient schedule to be administered in a LMIC setting in terms of PCV10 immunogenicity. We used both binding assays (ELISA) and functional assays (opsonophagocytic assay). These responses have been shown to correlate with protection against invasive pneumococcal disease, although the exact nature of that correlation remains unclear and probably varies by serotype.^21^ Our study design enabled us to make some key dosing schedule comparisons in infants in Vietnam. The first was a comparison of a two-dose (at ages 2 and 4 months) and three-dose (at ages 2, 3, and 4 months) primary vaccination series. Consistent with results of a systematic review^9^ and a trial in Nepal,^11^ we found that a two-dose or three-dose primary vaccination series yielded similar proportions of participants with protective levels of antibody for all serotypes except 6B and 23F (which were lower after two doses than after three doses), but higher IgG GMCs were seen after three doses than after two doses for most serotypes. Therefore, our overall criteria for non-inferiority (if found for at least seven of ten serotypes) in relation to the proportion of participants who had protective levels of antibodies was met. However, the true correlate of protection varies by serotype, and a higher concentration of antibody might be required to protect against mucosal diseases. Therefore, the difference in GMCs suggest that early protection might be reduced with a two-dose primary vaccination series, although the GMCs with this schedule were generally still high and more than half the GMC values seen after a three-dose primary series for all serotypes except 18C. Of all serotypes, serotypes 6B and 23F are affected the most by a reduction in the number of doses, although some studies have found that the concentration of antibody required to protect against those serotypes might be lower than for other serotypes, reducing this concern.^21^, ^22^ Opsonophagocytic assay results were similar to ELISA results; GMOIs were higher after a three-dose primary vaccination series for most serotypes (all except 4 and 19F) than after a two-dose primary vaccination series. Our data are consistent with the PCV10 trial in South Africa, which also found that three doses resulted in higher responses than two doses for several serotypes (six of ten serotypes by ELISA and five of these six by opsonophagocytic assay).^12^ However, the individual serotypes were different compared with our study, possibly due to variation in maternal antibody levels, reflecting local pneumococcal epidemiology in these two populations.

Before booster vaccination, antibody concentrations were still higher after a three-dose than a two-dose primary vaccination series for all serotypes except 19F. After booster vaccination, the 3 + 1 schedule resulted in higher antibody levels by ELISA for eight of ten serotypes (all except 19F and 6B) and higher functional antibody levels by opsonophagocytic assay for five of ten serotypes than the 2 + 1 schedule. However, at this timepoint almost all children in both groups had ELISA antibody levels of 0·35 μg/mL or higher and over 90% had an OI of 8 or higher, with no differences seen between those who had been given a two-dose or three-dose primary vaccination series. In the South African study, lower antibody levels were also seen before booster vaccination with a two-dose primary vaccination series than in a three-dose primary series, but similar levels were seen after booster vaccination both by ELISA and by opsonophagocytic assay for all serotypes except 7F and 14).^12^ Notably, over 60% of participants in our study had an OI of 8 or higher for serotypes 6A and 19A after the booster dose of PCV10. Schedules containing a booster dose provided higher levels of antibodies up to age 18 months than those without a booster dose, which might be important for protection into the second year of life.

Opsonophagocytic responses to serotype 1 are of particular interest, because of the importance of this serotype in LMICs,^23^ and the paucity of protection seen for serotype 1 with an investigational 9-valent PCV administered in 3 + 0 schedules in studies in The Gambia^24^ and South Africa.^25^ We found that although protective IgG responses to serotype 1 after a two-dose or three-dose primary series were high (>97%), opsonophagocytic responses were not as high, particularly after a two-dose primary series (66% of participants had an OI of ≥8). After the booster dose, the proportion of participants with protective levels of OI and IgG were high with both the 2 + 1 and 3 + 1 schedules, whereas, by contrast, OIs remained poor with the 3 + 0 schedule at age 10 months. This finding raises doubts about the protective efficacy of the 3 + 0 schedule against serotype 1, a matter of considerable importance in Africa, and raises the importance of a booster dose to maintain protection.^26^ Indeed, the implementation of PCV13 vaccination in a 2 + 1 schedule in South Africa has seen incidences of serotype 1 invasive pneumococcal disease reduced.^27^

Inclusion of the two-dose (2 and 6 months) group enabled us to assess a gap of 4 months between doses in a two-dose primary vaccination series compared with a gap of 2 months (the 2 + 1 schedule at ages 2, 4, and 9·5 months). For the problematic serotypes 6B and 23F, we found that a longer interval between doses led to more than 90% of infants having protective IgG concentrations compared with approximately 77% for the shorter interval. OIs of 8 or higher were also improved with the longer interval two-dose schedule for serotypes 1, 6B, 9V, 23F, and 6A. To our knowledge, this is the first assessment of this schedule, and the higher immune responses than with the short interval schedule are noteworthy. However, we recognise that participants were 2 months older at the time of sample collection (sample collected at 7 months *vs* sample collected at 5 months for the shorter interval schedule), so this finding could also be the result of a more mature immune system or a higher exposure to circulating serotypes. Nevertheless, our results suggest that protection could be maximised with an increased interval of more than 2 months between the first two doses of PCV10, recognising that during this interval there might be an increased risk of disease. The two-dose schedule at ages 2 and 6 months also produced surprisingly good results compared with post-booster responses after a 2 + 1 schedule, despite participants being 3 months younger (age 7 months compared with age 10·5 months). Similar GMCs were seen for three of ten vaccine serotypes (4, 5, and 19F), and the GMC values in the two-dose group were more than half the GMC values in the 2 + 1 group for all serotypes except 6B. Likewise, similar functional antibody levels (GMOIs) were seen for five of ten vaccine serotypes (4, 5, 7F, 9V, and 14) and the value of the GMOIs in the two-dose group were more than half the values in the 2 + 1 group for all serotypes except 18C. Given these results, hypothetically, a PCV schedule that incorporates both a wider primary series interval and a booster dose could provide better protection in the second year of life than current schedules. A two-dose schedule of PCV13 at ages 3 and 12 months has been introduced in the UK on the basis of promising immunogenicity data.^28^ The use of such a schedule in many LMICs, where coverage might be inadequate and vaccine types remain in circulation, might leave infants at risk of pneumococcal disease through the first year of life. One option to address this potential increased risk could be to bring the second dose forward to age 6 months, which for countries endemic for malaria would coincide with administration of the first dose of RTS,S malaria vaccine. Such schedules warrant further investigation.

The limitations of this study have been described previously^14^ and include the absence of clinical endpoints to assess these schedules and the potential for chance findings arising from the multiple comparisons done. Given the long history of PCV use and documented efficacy, our immunological assessment is appropriate and indeed a strength of this study because it enables inclusion of four different PCV10 schedules, including the two schedules recommended by WHO. We used both ELISA and opsonophagocytic assay at multiple timepoints to provide insight into the potential benefits and shortcomings of these schedules. We used a single a priori definition to determine non-inferiority, which was based on the proportion of infants with serotype-specific IgG concentrations of 0·35 μg/mL or higher, to define an overall pattern of responsiveness for each schedule and to compensate for the assessment of responses to multiple serotypes at several timepoints. Beyond this overall definition for non-inferiority, we made no formal adjustments for multiple comparisons.

Although direct protection against pneumococcal disease is important, so too is indirect protection though herd immunity and reduction of nasopharyngeal carriage.^29^ Analyses of the effect of these different PCV10 schedules on carriage and on memory B cells are ongoing.

In summary, we have shown a two-dose primary vaccination series to be non-inferior to a three-dose primary series, although lower antibody levels were seen by both ELISA and opsonophagocytic assay for most serotypes with the two-dose schedule than the three-dose schedule. For some serotypes, most notably serotype 1, a booster dose was necessary to generate strong opsonophagocytic responses, supporting the use of a 2 + 1 schedule when a total of three doses is to be administered. Two doses with a wider interval (at ages 2 and 6 months) resulted in higher antibody levels than the 2 + 1 schedule (at aged 2, 4, and 9·5 months) after the primary vaccination series, and was similar for several serotypes after booster vaccination. Considering all the results from the different schedules assessed in this study, the most efficient way to administer PCVs might be a schedule that is not currently in use anywhere in the world. The use of a two-dose primary series with a wider dosing interval of 4 months seems to provide similar or better protection after the primary vaccination series than a three-dose primary series, and enables a third dose to be given as a booster, thereby prolonging protection into the second year of life. Such a schedule might be especially beneficial in high burden settings.

## Data sharing

The study protocol and informed consent form have been published and are freely available.^15^ Data will be made publicly available in accordance with the rules set out by the Bill & Melinda Gates Foundation. Requests can be made by contacting the corresponding author.

## Declaration of interests

We declare no competing interests.
