# Supplementary material for: Immunogenicity of alternative ten-valent pneumococcal conjugate vaccine schedules in infants in Ho Chi Minh City, Vietnam: results from a single-blind, parallel-group, open-label, randomised, controlled trial
Source: Lancet Infect Dis. 2021 Oct;21(10):1415–28. doi: 10.1016/S1473-3099(20)30775-1 (PMC8461081; doi:10.1016/S1473-3099(20)30775-1)
Supplement: Supplementary appendix [file mmc1.pdf]

THE LANCET
Infectious Diseases

Supplementary appendix

This appendix formed part of the original submission and has been peer reviewed. We post it as supplied by the authors.

Supplement to: Licciardi PV, Temple B, Dai VTT, et al. Immunogenicity of alternative ten-valent pneumococcal conjugate vaccine schedules in infants in Ho Chi Minh City, Vietnam: results from a single-blind, parallel-group, open-label, randomised, controlled trial. *Lancet Infect Dis* 2021; published online June 22. https://doi.org/10.1016/S1473-3099(20)30775-1.

LIST OF APPENDIX TABLES AND FIGURES

Table S1: Timing of vaccinations and blood draws 2

Table S2: Baseline characteristics by study group 3

Figure S1: Comparative immunogenicity of PCV10 when administered according to different primary series schedules at 4 weeks post-primary series. 4

Table S3: Post-primary series immunogenicity of PCV10 in the intention-to-treat population. 5

Figure S2: Comparison of PCV10 immunogenicity post-primary series between infants who received a two doses at 2, 4 months or 2, 6 months (top panel) or between infants who received two doses at 2, 6 months or a 2+1 schedule at 2, 4, 9 months (bottom panel). 6

Table S4: Pre- and post-booster responses 7

Table S5: Antibody levels at 18 months of age 8

Figure S3: Comparison of serotype-specific IgG and OPA responses in the A) 3+0 and B) 2+1 PCV10 schedules. 9

Table S6: Comparative immunogenicity of a two-dose schedule (at 2 and 6 months of age) and a 2+1 schedule (at 2, 4, and 9 months of age) by ELISA and OPA 10

Table S1: Timing of vaccinations and blood draws

	Group A	Group B	Group C	Group D
PCV10 schedule	2, 3, 4 & 9 months (3+1)	2, 3 & 4 months (3+0)	2, 4 & 9.5* months (2+1)	2 & 6 months (two-dose)
Co-administered vaccines:				
DTP-Hib-HBV-IPV	2, 3, 4 & 19 months	2, 3, 4 & 19 months	2, 4, 9.5* & 19 months	2, 4, 6 & 19 months
measles	9 & 18 months	9 & 18 months	9 & 18 months	9 & 18 months
Blood samples:				
4-weeks post-one dose				3 months
4-weeks post-primary series	5 months	5 months	5 months	7 months
6 months of age		6 months	6 months (subset)†	6 months
Pre-booster/9 months	9 months	9 months (subset)†	9 months	9 months (subset)†
4-weeks post-booster/10 months	10 months	10 months	10 months	
18 months of age	18 months (subset)†	18 months (subset)†	18 months (subset)†	18 months (subset)†

PCV10 = 10-valent pneumococcal conjugate vaccine. DTP-Hib-HBV-IPV = hexavalent diphtheria, tetanus, pertussis, polio, *Haemophilus influenzae* type b, and hepatitis B vaccine.
* The booster doses of PCV10 and DTP-Hib-HBV-IPV in Group C were administered at 9.5 months of age as the Vietnamese Ministry of Health does not permit co-administration of measles and DTaP-IPV-Hib-HepB. † Participants allocated to Groups A–D from the last 300 recruited provided a blood sample at 18 months of age, with the remainder providing a sample at an alternative time point, as a maximum of 4 blood samples per participant were permitted by the Vietnamese Ministry of Health.

Table S2: Baseline characteristics by study group

	Group A (n=152)	Group B (n=149)	Group C (n=250)	Group D (n=202)
Sex				
Male	66 (43%)	73 (49%)	135 (54%)	91 (45%)
Female	86 (57%)	76 (51%)	115 (46%)	111 (55%)
District				
4	68 (45%)	67 (45%)	112 (45%)	90 (45%)
7	84 (55%)	82 (55%)	138 (55%)	112 (55%)
Birthweight (g)*	3234 (424)	3212 (349)	3228 (370)	3234 (410)
Place of delivery				
Hospital	149 (98%)	149 (100%)	245 (98%)†	194 (96%)
Other	3 (2%)	0 (0%)	4 (2%)	8 (4%)
Type of delivery				
Normal	89 (59%)	85 (57%)	160 (64%)	130 (64%)
Elective caesarean	30 (20%)	30 (20%)	43 (17%)	36 (18%)
Emergency caesarean	27 (18%)	30 (20%)	40 (16%)	34 (17%)
Other/unknown	6 (4%)	4 (3%)	7 (3%)	2 (1%)
Cigarette smoker in house				
No	57 (38%)	52 (35%)	81 (33%)†	74 (37%)
Yes	95 (62%)	97 (65%)	168 (67%)	128 (63%)
Breastfeeding at enrolment				
No	41 (27%)†	42 (28%)	55 (22%)	37 (18%)
Yes	110 (73%)	107 (72%)	195 (78%)	165 (82%)

Data are n(%) or mean(SD).
* Birthweight data missing for 7 participants (1, 3, and 3 from Groups B, C, and D, respectively).
† Data missing for one participant.

Figure S1: Comparative immunogenicity of PCV10 when administered according to different primary series schedules at 4 weeks post-primary series.
The data are differences in the proportions of infants with protective serotype-specific IgG concentrations (≥0·35μg/mL) (A) Three-dose primary series of PCV10 (at 2, 3, 4 months; combined Groups A+B) versus two-dose primary series of PCV10 (at 2, 4 months; Group C). (B) Two-dose primary series of PCV10 (Group C) given at 2, 4 months compared with a two-dose primary series of PCV10 (Group D) given at 2, 6 months. Bars represent 90% CIs for one-sided tests of non-inferiority (A) or 95% Cis for two-sided tests of difference (B), with solid vertical lines indicating the predefined thresholds for determining non-inferiority or differences between groups.

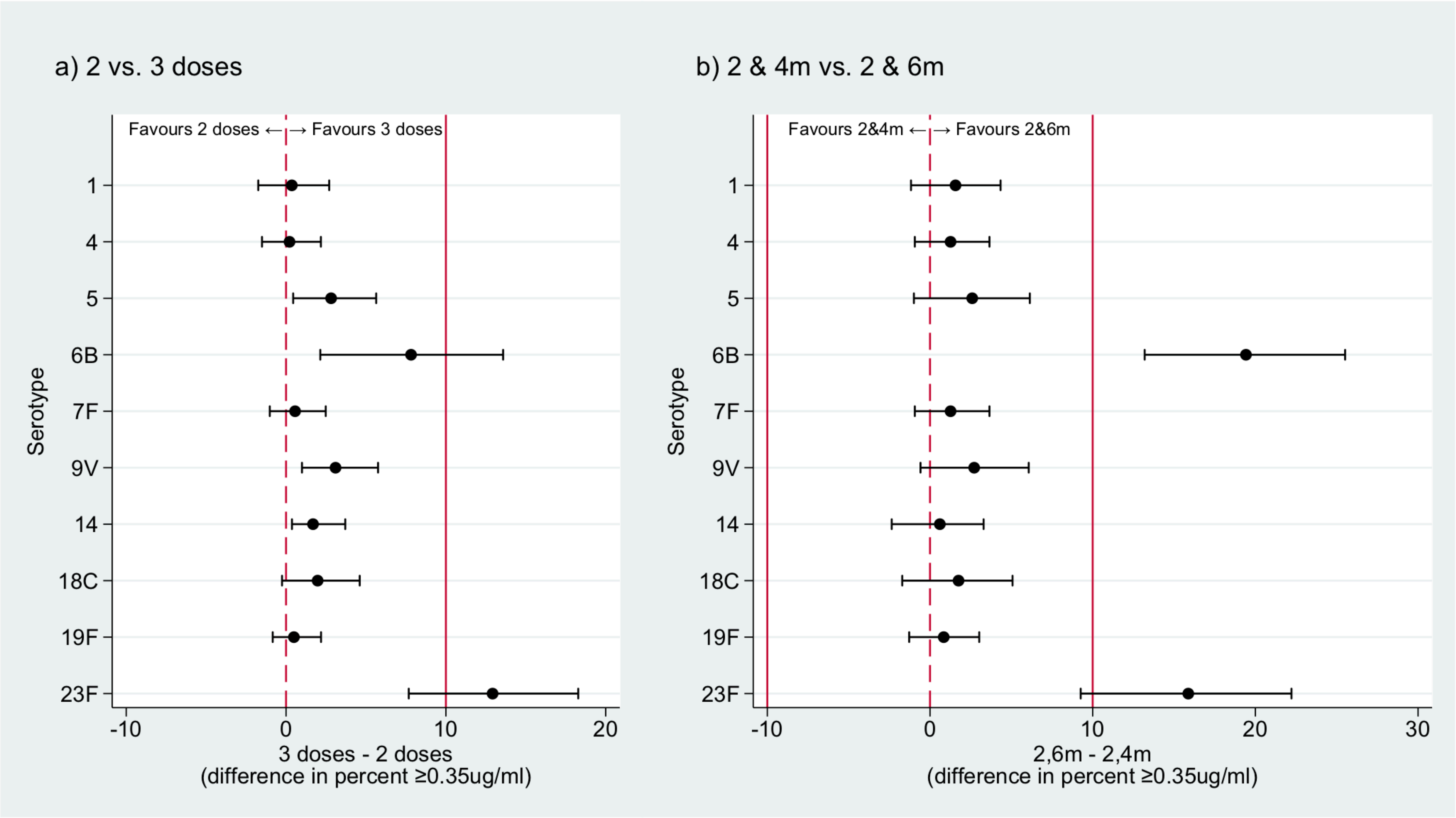

Table S3: Post-primary series immunogenicity of PCV10 in the intention-to-treat population.

	Participants with IgG concentration ≥0·35µg/ml, % (95% CI)			Risk difference, %		GMC, µg/ml (95% CI)			GMC ratio (95% CI)	
	2,3,4m (n=289)	2,4m (n=240)	2,6m (n=195)	2,3,4m minus 2,4m (90% CI)	2,6m minus 2,4m (95% CI)	2,3,4m (n=289)	2,4m (n=240)	2,6m (n=195)	2,3,4m / 2,4m	2,6m / 2,4m
Vaccine serotypes										
1	98.3 (96.0, 99.4)	97.9 (95.2, 99.3)	99.5 (97.2, 100)	0.4 (-1.7, 2.7)	1.6 (-1.0, 4.3)	2.78 (2.51, 3.08)	2.22 (1.98, 2.48)	3.30 (2.93, 3.72)	1.25 (1.08, 1.46)	1.49 (1.26, 1.75)
4	99.0 (97.0, 99.8)	98.8 (96.4, 99.7)	100 (98.1, 100)	0.2 (-1.5, 2.2)	1.3 (-0.9, 3.6)	3.85 (3.45, 4.30)	3.21 (2.88, 3.58)	4.20 (3.76, 4.68)	1.20 (1.02, 1.40)	1.31 (1.12, 1.53)
5	98.6 (96.5, 99.6)	95.8 (92.5, 98.0)	98.5 (95.6, 99.7)	2.8 (0.4, 5.6)	2.6 (-0.8, 6.1)	1.82 (1.67, 1.98)	1.17 (1.07, 1.27)	1.28 (1.17, 1.39)	1.55 (1.38, 1.75)	1.09 (0.97, 1.23)
6B	84.4 (79.7, 88.4)	77.1 (71.2, 82.2)	96.4 (92.7, 98.5)	7.3 (1.7, 13.1)	19.3 (13.3, 25.3)	1.08 (0.95, 1.23)	0.80 (0.70, 0.92)	2.62 (2.29, 3.00)	1.34 (1.11, 1.62)	3.26 (2.69, 3.96)
7F	99.3 (97.5, 99.9)	98.8 (96.4, 99.7)	99.5 (97.2, 100)	0.6 (-1.0, 2.5)	0.7 (-1.7, 3.1)	3.03 (2.78, 3.30)	2.07 (1.89, 2.26)	2.16 (1.97, 2.36)	1.47 (1.30, 1.66)	1.05 (0.92, 1.19)
9V	99.3 (97.5, 99.9)	96.3 (93.0, 98.3)	99.0 (96.3, 99.9)	3.1 (1.0, 5.7)	2.7 (-0.4, 6.0)	2.47 (2.26, 2.70)	1.63 (1.47, 1.81)	2.37 (2.14, 2.62)	1.52 (1.32, 1.74)	1.45 (1.26, 1.68)
14	100 (98.7, 100)	98.3 (95.8, 99.5)	99.0 (96.3, 99.9)	1.7 (0.4, 3.7)	0.6 (-2.2, 3.3)	9.72 (8.76, 10.79)	5.92 (5.17, 6.78)	8.57 (7.45, 9.85)	1.64 (1.39, 1.94)	1.45 (1.19, 1.76)
18C	98.6 (96.5, 99.6)	96.7 (93.5, 98.6)	98.5 (95.6, 99.7)	1.9 (-0.3, 4.5)	1.8 (-1.5, 5.1)	3.86 (3.47, 4.29)	1.87 (1.65, 2.12)	2.93 (2.60, 3.30)	2.06 (1.76, 2.43)	1.57 (1.32, 1.87)
19F	99.7 (98.1, 100)	99.2 (97.0, 99.9)	100 (98.1, 100)	0.5 (-0.8, 2.2)	0.8 (-1.2, 3.0)	8.22 (7.42, 9.12)	9.56 (8.40, 10.88)	16.15 (14.27, 18.28)	0.86 (0.73, 1.01)	1.69 (1.41, 2.02)
23F	90.3 (86.3, 93.5)	77.9 (72.1, 83.0)	93.8 (89.5, 96.8)	12.4 (7.2, 17.7)	15.9 (9.5, 22.2)	1.32 (1.18, 1.48)	0.89 (0.78, 1.02)	2.16 (1.90, 2.46)	1.48 (1.24, 1.76)	2.43 (2.01, 2.93)
Cross-reacting serotypes										
6A	50.5 (44.6, 56.4)	40.8 (34.6, 47.3)	70.3 (63.3, 76.6)	9.7 (2.5, 16.7)	29.4 (20.2, 37.9)	0.37 (0.34, 0.41)	0.31 (0.28, 0.35)	0.65 (0.57, 0.74)	1.18 (1.02, 1.36)	2.07 (1.74, 2.46)
19A	67.8 (62.1, 73.2)	70.0 (63.8, 75.7)	85.6 (79.9, 90.2)	-2.2 (-8.7, 4.5)	15.6 (7.8, 23.0)	0.56 (0.50, 0.62)	0.55 (0.49, 0.61)	0.93 (0.81, 1.06)	1.02 (0.88, 1.19)	1.70 (1.42, 2.02)

Immunogenicity data 4-weeks after three doses (at 2, 3, and 4 months of age, Group A+B), two doses (at 2 and 4 months of age, Group C), or two doses (at 2 and 6 months of age, Group D).

Figure S2: Comparison of PCV10 immunogenicity post-primary series between infants who received a two doses at 2, 4 months or 2, 6 months (top panel) or between infants who received two doses at 2, 6 months or a 2+1 schedule at 2, 4, 9 months (bottom panel).
Geometric mean ratios for serotype-specific IgG (A) and opsonophagocytosis (B) are shown with 95% CIs. Comparisons were made at 5 months of age (2, 4m schedule), 7 months of age (2, 6m schedule) or 10 months of age (2, 4, 9m schedule). GMC=geometric mean concentration; GMOI=geometric mean opsonisation index.

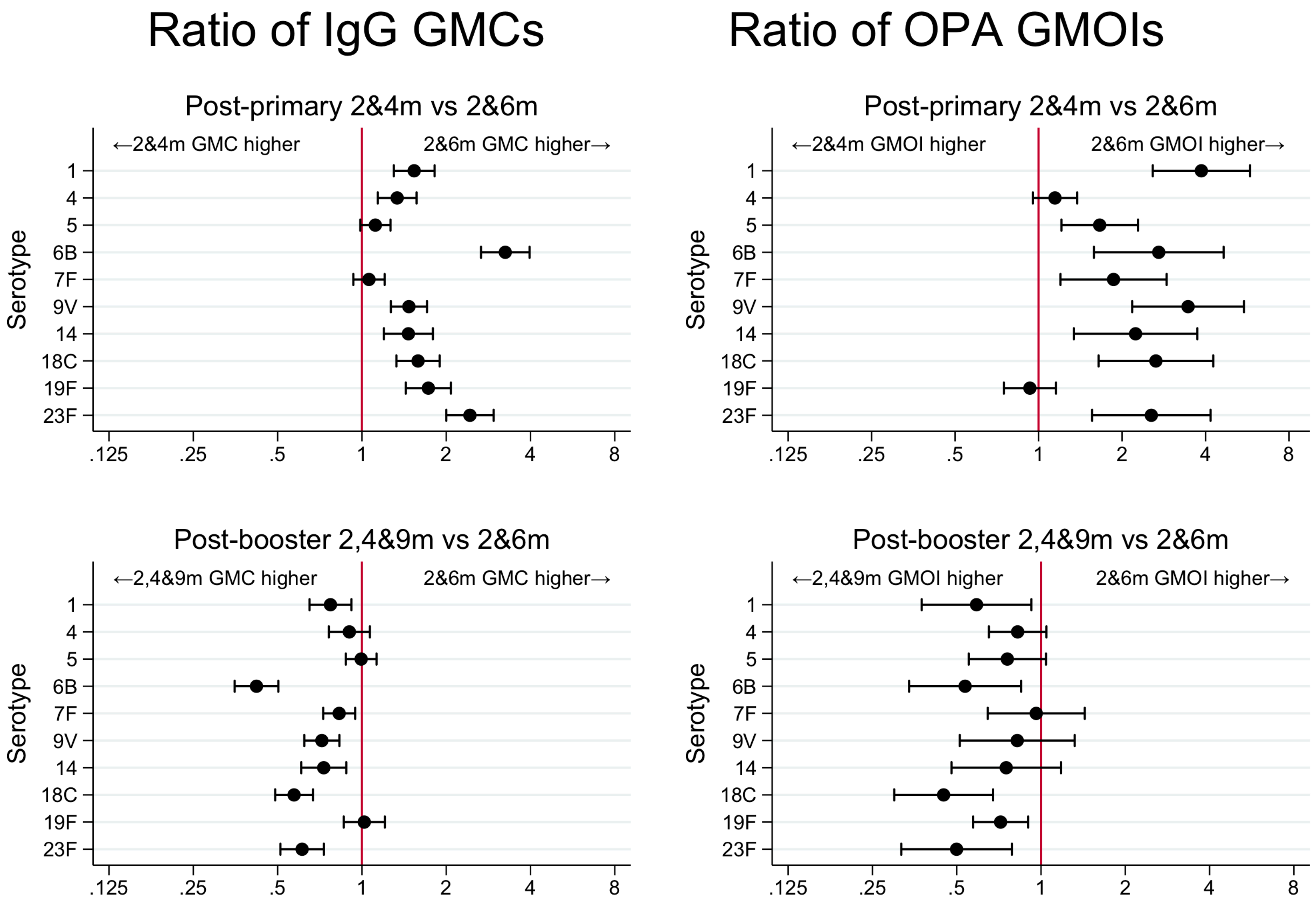

Table S4: Pre- and post-booster responses

a) IgG GMCs (95% CI) pre- and post-booster in a 3+1 or 2+1 PCV10 schedule, or at 9 and 10 months of age in a 3+0 PCV10 schedule

	3+1		2+1		3+0		GMC ratio (95% CI)	
	Pre-booster (n=145)	Post-booster (n=137)	Pre-booster (n=236)	Post-booster (n=226)	9m (n=90)	10m (n=133)	Pre-booster (3+1 / 2+1)	Post-booster (3+1 / 2+1)
Vaccine serotypes								
1	0.90 (0.79, 1.03)	5.85 (4.92, 6.96)	0.71 (0.64, 0.79)	4.40 (3.91, 4.97)	0.94 (0.79, 1.12)	0.78 (0.67, 0.91)	1.26 (1.07, 1.49)	1.33 (1.08, 1.63)
4	1.38 (1.20, 1.59)	6.06 (5.13, 7.16)	1.09 (0.98, 1.22)	4.75 (4.20, 5.37)	1.43 (1.19, 1.73)	1.16 (0.99, 1.36)	1.27 (1.06, 1.51)	1.28 (1.04, 1.56)
5	0.77 (0.69, 0.86)	1.96 (1.74, 2.22)	0.54 (0.49, 0.59)	1.31 (1.20, 1.43)	0.71 (0.61, 0.83)	0.68 (0.60, 0.78)	1.44 (1.25, 1.67)	1.50 (1.29, 1.73)
6B	2.23 (1.97, 2.51)	7.42 (6.38, 8.63)	1.63 (1.44, 1.83)	6.17 (5.50, 6.92)	2.34 (1.94, 2.83)	2.14 (1.83, 2.52)	1.37 (1.14, 1.64)	1.20 (1.00, 1.45)
7F	1.23 (1.09, 1.40)	4.53 (3.93, 5.22)	0.83 (0.76, 0.91)	2.65 (2.41, 2.91)	1.19 (1.01, 1.40)	0.95 (0.82, 1.10)	1.48 (1.27, 1.74)	1.71 (1.45, 2.01)
9V	1.14 (1.01, 1.30)	4.67 (4.09, 5.34)	0.75 (0.68, 0.84)	3.34 (3.02, 3.69)	1.18 (1.00, 1.39)	1.07 (0.92, 1.25)	1.52 (1.29, 1.79)	1.40 (1.19, 1.65)
14	5.91 (5.09, 6.86)	16.14 (13.72, 18.98)	3.41 (2.96, 3.94)	11.76 (10.45, 13.24)	6.74 (5.44, 8.34)	5.59 (4.63, 6.74)	1.73 (1.39, 2.15)	1.37 (1.13, 1.67)
18C	1.06 (0.93, 1.21)	6.65 (5.76, 7.67)	0.81 (0.72, 0.90)	5.16 (4.68, 5.70)	1.13 (0.95, 1.36)	0.89 (0.76, 1.02)	1.32 (1.11, 1.57)	1.29 (1.09, 1.52)
19F	3.70 (3.30, 4.15)	16.26 (14.04, 18.83)	3.94 (3.59, 4.31)	16.16 (14.45, 18.08)	4.00 (3.35, 4.78)	3.39 (2.91, 3.95)	0.94 (0.81, 1.09)	1.01 (0.84, 1.21)
23F	1.01 (0.89, 1.14)	4.81 (4.18, 5.54)	0.76 (0.68, 0.86)	3.55 (3.15, 3.99)	1.25 (1.02, 1.53)	1.03 (0.86, 1.23)	1.32 (1.10, 1.58)	1.36 (1.12, 1.63)
Cross-reacting serotypes								
6A	0.67 (0.58, 0.77)	1.62 (1.32, 1.98)	0.57 (0.51, 0.65)	1.44 (1.25, 1.66)	0.81 (0.65, 1.00)	0.75 (0.63, 0.89)	1.16 (0.96, 1.40)	1.12 (0.88, 1.43)
19A	0.67 (0.59, 0.75)	1.75 (1.48, 2.05)	0.66 (0.60, 0.73)	1.76 (1.55, 2.00)	0.86 (0.71, 1.04)	0.80 (0.69, 0.93)	1.01 (0.87, 1.18)	0.99 (0.81, 1.22)

b) Percentage of participants with IgG ≥0·35µg/ml (95% CI) pre- and post-booster in a 3+1 or 2+1 PCV10 schedule, or at 9 and 10 months of age in a 3+0 PCV10 schedule

	3+1		2+1		3+0		Risk difference, % (95% CI)	
	Pre-booster (n=145)	Post-booster (n=137)	Pre-booster (n=236)	Post-booster (n=226)	9m (n=90)	10m (n=133)	Pre-booster (3+1 minus 2+1)	Post-booster (3+1 minus 2+1)
Vaccine serotypes								
1	91.7 (86.0, 95.7)	100 (97.3, 100)	82.6 (77.2, 87.2)	100 (98.4, 100)	90.0 (81.9, 95.3)	86.5 (79.5, 91.8)	9.1 (2.0, 15.5)	0.0 (-2.7, 1.7)
4	97.9 (94.1, 99.6)	100 (97.3, 100)	91.1 (86.7, 94.4)	98.7 (96.2, 99.7)	94.4 (87.5, 98.2)	91.0 (84.8, 95.3)	6.8 (2.0, 11.4)	1.3 (-1.5, 3.8)
5	89.7 (83.5, 94.1)	100 (97.3, 100)	75.4 (69.4, 80.8)	97.8 (94.9, 99.3)	86.7 (77.9, 92.9)	82.0 (74.4, 88.1)	14.2 (6.4, 21.3)	2.2 (-0.8, 5.1)
6B	98.6 (95.1, 99.8)	100 (97.3, 100)	94.9 (91.3, 97.3)	100 (98.4, 100)	97.8 (92.2, 99.7)	94.7 (89.5, 97.9)	3.7 (-0.4, 7.4)	0.0 (-2.7, 1.7)
7F	95.2 (90.3, 98.0)	100 (97.3, 100)	90.3 (85.7, 93.7)	99.6 (97.6, 100)	95.6 (89.0, 98.8)	91.0 (84.8, 95.3)	4.9 (-0.8, 10.0)	0.4 (-2.3, 2.5)
9V	94.5 (89.4, 97.6)	100 (97.3, 100)	86.0 (80.9, 90.2)	100 (98.4, 100)	94.4 (87.5, 98.2)	91.0 (84.8, 95.3)	8.5 (2.2, 14.1)	0.0 (-2.7, 1.7)
14	100 (97.5, 100)	100 (97.3, 100)	97.5 (94.5, 99.1)	99.6 (97.6, 100)	100 (96.0, 100)	98.5 (94.7, 99.8)	2.5 (-0.4, 5.4)	0.4 (-2.3, 2.5)
18C	93.8 (88.5, 97.1)	100 (97.3, 100)	84.7 (79.5, 89.1)	100 (98.4, 100)	92.2 (84.6, 96.8)	89.5 (83.0, 94.1)	9.0 (2.5, 15.0)	0.0 (-2.7, 1.7)
19F	99.3 (96.2, 100)	100 (97.3, 100)	100 (98.4, 100)	100 (98.4, 100)	100 (96.0, 100)	99.2 (95.9, 100.0)	-0.7 (-3.8, 1.0)	0.0 (-2.7, 1.7)
23F	89.7 (83.5, 94.1)	100 (97.3, 100)	83.1 (77.6, 87.6)	98.7 (96.2, 99.7)	91.1 (83.2, 96.1)	88.0 (81.2, 93.0)	6.6 (-0.8, 13.2)	1.3 (-1.5, 3.8)
Cross-reacting serotypes								
6A	78.6 (71.0, 85.0)	92.7 (87.0, 96.4)	69.9 (63.6, 75.7)	91.6 (87.2, 94.9)	81.1 (71.5, 88.6)	80.5 (72.7, 86.8)	8.7 (-0.5, 17.2)	1.1 (-5.2, 6.6)
19A	81.4 (74.1, 87.4)	92.7 (87.0, 96.4)	78.8 (73.0, 83.8)	95.6 (92.0, 97.9)	83.3 (74.0, 90.4)	80.5 (72.7, 86.8)	2.6 (-6.0, 10.5)	-2.9 (-8.8, 1.9)

Table S5: Antibody levels at 18 months of age

a) IgG GMCs (95% CIs) at 18 months of age following a 3+1, 2+1, 3+0 or two-dose schedule of PCV10

	**GMC, µg/ml (95% CI)**				**Ratio of GMCs (95% CI)**		
	**3+1** (n=48)	**2+1** (n=47)	**3+0** (n=40)	**two-dose** (n=49)	**3+1 / 2+1**	**2+1 / 3+0**	**2+1 / two-dose**
Vaccine serotypes							
1	0.77 (0.58, 1.02)	0.68 (0.53, 0.87)	0.35 (0.24, 0.50)	0.29 (0.24, 0.36)	1.14 (0.79, 1.65)	1.95 (1.28, 2.95)	2.31 (1.70, 3.14)
4	0.70 (0.51, 0.96)	0.56 (0.45, 0.71)	0.33 (0.24, 0.47)	0.30 (0.24, 0.38)	1.23 (0.84, 1.81)	1.69 (1.14, 2.50)	1.87 (1.36, 2.57)
5	0.69 (0.57, 0.85)	0.61 (0.49, 0.74)	0.55 (0.44, 0.68)	0.47 (0.39, 0.57)	1.15 (0.87, 1.52)	1.11 (0.82, 1.49)	1.29 (0.97, 1.70)
6B	1.68 (1.21, 2.32)	1.15 (0.87, 1.54)	1.05 (0.73, 1.51)	0.78 (0.58, 1.05)	1.46 (0.95, 2.23)	1.10 (0.70, 1.72)	1.48 (0.99, 2.22)
7F	0.68 (0.53, 0.87)	0.46 (0.35, 0.59)	0.34 (0.25, 0.45)	0.29 (0.23, 0.37)	1.48 (1.04, 2.11)	1.36 (0.93, 1.98)	1.57 (1.11, 2.22)
9V	0.63 (0.49, 0.81)	0.55 (0.45, 0.67)	0.43 (0.32, 0.58)	0.32 (0.26, 0.39)	1.15 (0.84, 1.57)	1.29 (0.91, 1.82)	1.74 (1.31, 2.32)
14	2.92 (2.06, 4.14)	1.94 (1.49, 2.52)	1.63 (1.16, 2.27)	1.10 (0.76, 1.60)	1.51 (0.98, 2.32)	1.19 (0.79, 1.80)	1.76 (1.12, 2.76)
18C	0.85 (0.63, 1.13)	0.67 (0.53, 0.86)	0.30 (0.22, 0.41)	0.30 (0.24, 0.38)	1.26 (0.87, 1.83)	2.21 (1.51, 3.23)	2.22 (1.61, 3.05)
19F	3.92 (3.05, 5.05)	3.36 (2.56, 4.40)	1.85 (1.27, 2.68)	1.92 (1.56, 2.35)	1.17 (0.81, 1.68)	1.82 (1.16, 2.84)	1.75 (1.25, 2.44)
23F	0.96 (0.73, 1.24)	0.77 (0.59, 1.01)	0.55 (0.39, 0.78)	0.43 (0.33, 0.57)	1.24 (0.85, 1.81)	1.40 (0.91, 2.15)	1.77 (1.21, 2.60)
Cross-reacting serotypes							
6A	0.72 (0.55, 0.95)	0.59 (0.46, 0.76)	0.64 (0.46, 0.89)	0.49 (0.37, 0.65)	1.23 (0.85, 1.78)	0.92 (0.62, 1.38)	1.20 (0.83, 1.73)
19A	0.95 (0.76, 1.18)	0.86 (0.66, 1.13)	0.74 (0.53, 1.04)	0.64 (0.52, 0.80)	1.10 (0.78, 1.55)	1.16 (0.76, 1.78)	1.34 (0.95, 1.88)

b) Percentage of participants with IgG ≥0·35µg/ml (95% CI) at 18 months of age following a 3+1, 2+1, 3+0 or two-dose schedule of PCV10

	**Participants with IgG concentration ≥0·35µg/ml, % (95% CI)**				**Risk difference, % (95% CI)**		
	**3+1** (n=48)	**2+1** (n=47)	**3+0** (n=40)	**two-dose** (n=49)	**3+1 minus 2+1**	**2+1 minus 3+0**	**2+1 minus two-dose**
Vaccine serotypes							
1	75.0 (60.4, 86.4)	76.6 (62.0, 87.7)	42.5 (27.0, 59.1)	49.0 (34.4, 63.7)	-1.6 (-18.5, 15.5)	34.1 (13.5, 51.2)	27.6 (8.3, 44.2)
4	68.8 (53.7, 81.3)	72.3 (57.4, 84.4)	40.0 (24.9, 56.7)	44.9 (30.7, 59.8)	-3.6 (-21.3, 14.5)	32.3 (11.5, 49.7)	27.4 (7.7, 44.3)
5	89.6 (77.3, 96.5)	80.9 (66.7, 90.9)	70.0 (53.5, 83.4)	73.5 (58.9, 85.1)	8.7 (-5.9, 23.4)	10.9 (-7.1, 28.6)	7.4 (-9.5, 23.7)
6B	93.8 (82.8, 98.7)	95.7 (85.5, 99.5)	90.0 (76.3, 97.2)	85.7 (72.8, 94.1)	-2.0 (-13.0, 8.8)	5.7 (-5.9, 19.2)	10.0 (-2.3, 22.8)
7F	77.1 (62.7, 88.0)	59.6 (44.3, 73.6)	45.0 (29.3, 61.5)	34.7 (21.7, 49.6)	17.5 (-1.2, 34.7)	14.6 (-6.2, 33.8)	24.9 (4.9, 42.3)
9V	72.9 (58.2, 84.7)	83.0 (69.2, 92.4)	52.5 (36.1, 68.5)	42.9 (28.8, 57.8)	-10.1 (-26.2, 6.8)	30.5 (10.9, 47.5)	40.1 (21.0, 55.3)
14	95.8 (85.7, 99.5)	97.9 (88.7, 99.9)	97.5 (86.8, 99.9)	89.8 (77.8, 96.6)	-2.0 (-12.0, 7.4)	0.4 (-8.8, 10.9)	8.1 (-2.6, 19.8)
18C	83.3 (69.8, 92.5)	74.5 (59.7, 86.1)	35.0 (20.6, 51.7)	44.9 (30.7, 59.8)	8.9 (-7.6, 25.0)	39.5 (18.6, 55.9)	29.6 (9.9, 46.2)
19F	100 (92.6, 100)	100 (92.5, 100)	97.5 (86.8, 99.9)	98.0 (89.1, 99.9)	0.0 (-7.4, 7.6)	2.5 (-5.3, 12.9)	2.0 (-5.7, 10.7)
23F	87.5 (74.8, 95.3)	80.9 (66.7, 90.9)	60.0 (43.3, 75.1)	59.2 (44.2, 73.0)	6.6 (-8.4, 21.6)	20.9 (1.7, 38.6)	21.7 (3.3, 38.1)
Cross-reacting serotypes							
6A	75.0 (60.4, 86.4)	74.5 (59.7, 86.1)	82.5 (67.2, 92.7)	67.3 (52.5, 80.1)	0.5 (-16.7, 17.8)	-8.0 (-24.5, 9.7)	7.1 (-10.9, 24.5)
19A	91.7 (80.0, 97.7)	83.0 (69.2, 92.4)	77.5 (61.5, 89.2)	77.6 (63.4, 88.2)	8.7 (-5.2, 22.7)	5.5 (-11.1, 22.5)	5.4 (-10.7, 21.1)

Figure S3: Comparison of serotype-specific IgG and OPA responses in the A) 3+0 and B) 2+1 PCV10 schedules.
Bars represent the percentage of infants with IgG levels ≥0·35µg/mL or OI≥8, post-primary series (5m) or post-booster (10m) for serotypes 18C, 6B, 1 and 23F.

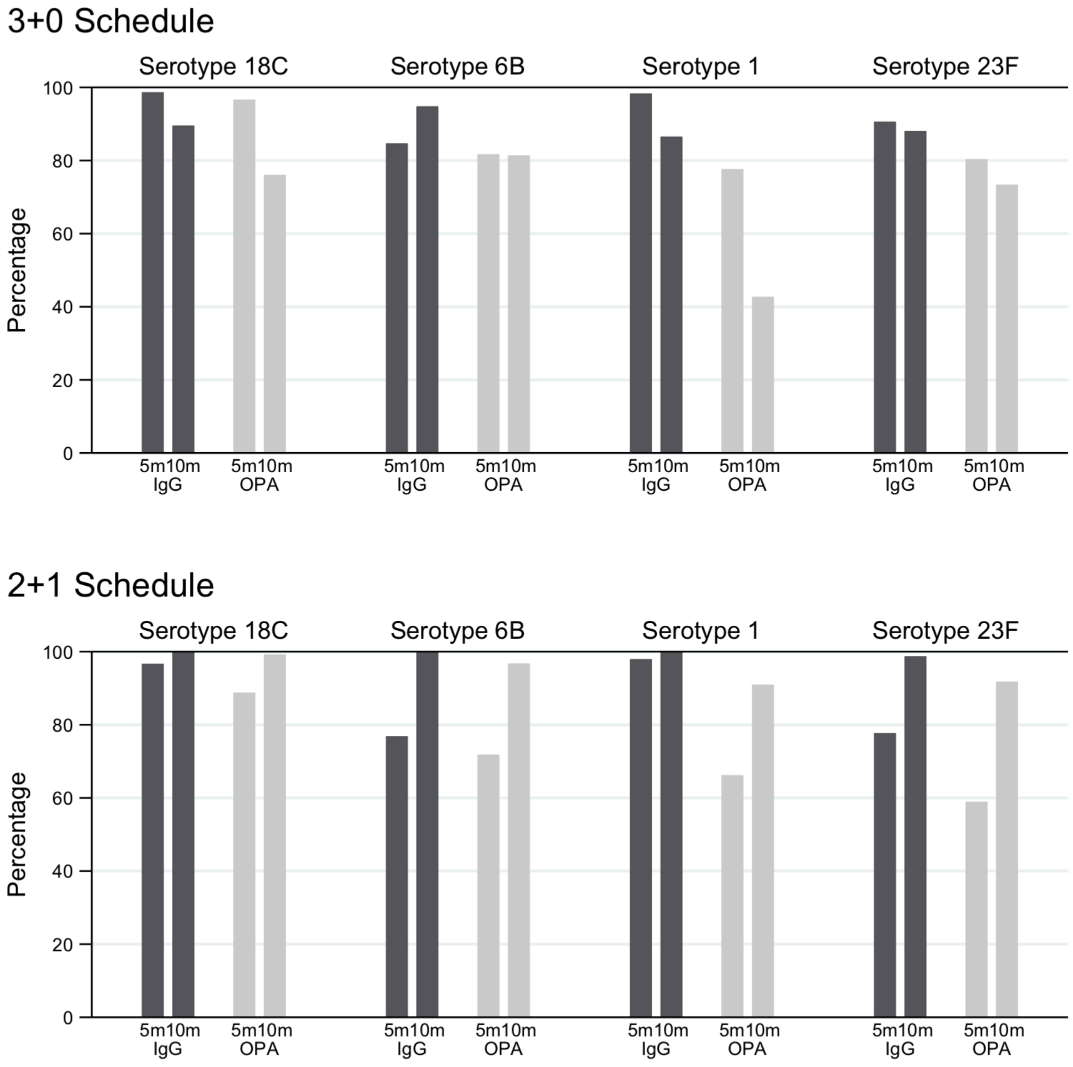

Table S6: Comparative immunogenicity of a two-dose schedule (at 2 and 6 months of age) and a 2+1 schedule (at 2, 4, and 9 months of age) by ELISA and OPA

	GMC, µg/ml (95% CI)			GMC ratio (95% CI)		Geometric mean opsonisation index (95% CI)			Ratio of geometric mean opsonisation indices (95% CI)	
	2,6m (n=185)	2,4m (n=237)	2,4&9m (n=226)	2,6m / 2,4m	2,6m / 2,4&9m	2,6m (n=97)	2,4m (n=124)	2,4&9m (n=121)	2,6m / 2,4m	2,6m / 2,4&9m
Vaccine serotypes										
1	3.40 (3.01, 3.84)	2.21 (1.97, 2.48)	4.40 (3.91, 4.97)	1.54 (1.30, 1.82)	0.77 (0.65, 0.92)	85 (62, 118)	22 (17, 28)	145 (106, 198)	3.86 (2.58, 5.79)	0.59 (0.38, 0.92)
4	4.28 (3.83, 4.79)	3.21 (2.87, 3.58)	4.75 (4.20, 5.37)	1.34 (1.14, 1.57)	0.90 (0.76, 1.07)	1056 (912, 1223)	922 (820, 1036)	1280 (1072, 1529)	1.15 (0.95, 1.38)	0.83 (0.65, 1.05)
5	1.31 (1.19, 1.43)	1.17 (1.07, 1.27)	1.31 (1.20, 1.43)	1.12 (0.99, 1.26)	0.99 (0.88, 1.13)	582 (453, 749)	351 (286, 430)	768 (627, 941)	1.66 (1.21, 2.28)	0.76 (0.55, 1.04)
6B	2.59 (2.25, 2.99)	0.80 (0.69, 0.92)	6.17 (5.50, 6.92)	3.25 (2.66, 3.97)	0.42 (0.35, 0.50)	160 (111, 233)	59 (40, 86)	299 (224, 399)	2.71 (1.58, 4.65)	0.54 (0.34, 0.85)
7F	2.20 (2.01, 2.40)	2.07 (1.89, 2.27)	2.65 (2.41, 2.91)	1.06 (0.93, 1.21)	0.83 (0.73, 0.95)	466 (348, 623)	250 (182, 343)	484 (369, 636)	1.86 (1.20, 2.90)	0.96 (0.65, 1.43)
9V	2.40 (2.17, 2.66)	1.63 (1.47, 1.81)	3.34 (3.02, 3.69)	1.47 (1.27, 1.71)	0.72 (0.62, 0.83)	253 (186, 343)	73 (52, 102)	308 (217, 436)	3.46 (2.18, 5.50)	0.82 (0.51, 1.32)
14	8.59 (7.43, 9.94)	5.86 (5.11, 6.73)	11.76 (10.45, 13.24)	1.47 (1.20, 1.79)	0.73 (0.61, 0.88)	296 (210, 417)	132 (92, 191)	394 (293, 531)	2.24 (1.34, 3.74)	0.75 (0.48, 1.18)
18C	2.95 (2.61, 3.34)	1.86 (1.64, 2.11)	5.16 (4.68, 5.70)	1.59 (1.33, 1.89)	0.57 (0.49, 0.67)	329 (239, 453)	124 (88, 175)	732 (564, 950)	2.65 (1.65, 4.26)	0.45 (0.30, 0.67)
19F	16.47 (14.50, 18.72)	9.54 (8.37, 10.87)	16.16 (14.45, 18.08)	1.73 (1.43, 2.08)	1.02 (0.86, 1.21)	1133 (936, 1371)	1217 (1078, 1375)	1579 (1380, 1807)	0.93 (0.75, 1.16)	0.72 (0.57, 0.90)
23F	2.17 (1.90, 2.48)	0.89 (0.78, 1.02)	3.55 (3.15, 3.99)	2.43 (2.00, 2.95)	0.61 (0.51, 0.73)	74 (53, 104)	29 (21, 41)	149 (109, 202)	2.55 (1.56, 4.17)	0.50 (0.32, 0.79)
Cross-reacting serotypes										
6A	0.64 (0.55, 0.73)	0.31 (0.28, 0.35)	1.44 (1.25, 1.66)	2.04 (1.71, 2.43)	0.44 (0.36, 0.54)	77 (46, 130)	18 (12, 26)	118 (74, 189)	4.39 (2.31, 8.33)	0.65 (0.32, 1.31)
19A	0.93 (0.81, 1.06)	0.55 (0.49, 0.62)	1.76 (1.55, 2.00)	1.68 (1.41, 2.01)	0.53 (0.44, 0.64)	16 (11, 23)	9 (7, 11)	25 (18, 34)	1.90 (1.29, 2.81)	0.66 (0.41, 1.05)

Immunogenicity 4-weeks after a two-dose schedule (blood at 7 months of age), compared with immunogenicity 4-weeks post-primary series (blood at 5 months of age) and immunogenicity 4-weeks post-booster (blood at 10 months of age) in a 2+1 schedule.
